# Discovery of a Novel Bloom’s Syndrome Protein (BLM) Inhibitor Suppressing Growth and Metastasis of Prostate Cancer

**DOI:** 10.3390/ijms232314798

**Published:** 2022-11-26

**Authors:** Xiao-Yan Ma, Hou-Qiang Xu, Jia-Fu Zhao, Yong Ruan, Bin Chen

**Affiliations:** 1 Key Laboratory of Animal Genetics, Breeding and Reproduction in the Plateau Mountainous Region, Ministry of Education, College of Life Sciences, Guizhou University, Guiyang 550025, China; 2 College of Food and Pharmaceutical Engineering, Guizhou Institute of Technology, Guiyang 550003, China; 3 College of Animal Science, Guizhou University, Guiyang 550025, China

**Keywords:** BLM, AO/854, prostate cancer, cell proliferation, cell apoptosis

## Abstract

Prostate cancer (PCa) is a common cancer and a major cause of cancer-related death worldwide in men, necessitating novel targets for cancer therapy. High expression of Bloom’s syndrome protein (BLM) helicase is associated with the occurrence and development of PCa. Therefore, the identification and development of new BLM inhibitors may be a new direction for the treatment of PCa. Here, we identified a novel inhibitor by molecular docking and put it to systematic evaluation via various experiments, AO/854, which acted as a competitive inhibitor that blocked the BLM-DNA interaction. Cellular evaluation indicated that AO/854-suppressed tumor growth and metastasis in PC3 cells by enhancing DNA damage, phosphorylating Chk1/Chk2, and altering the p53 signaling pathway. Collectively, the study highlights the potential of BLM as a therapeutic target in PCa and reveals a distinct mechanism by which AO/854 competitively inhibits the function of BLM.

## 1. Introduction

Prostate cancer (PCa) affects over 1.3 million people worldwide each year, making it a serious health concern [1]. A diagnosis of PCa affects about 10 million men worldwide, and 700,000 of these individuals are living with metastatic disease [2,3]. Many factors contribute to PCa development, including age, diet, and genetics [4,5,6,7,8]. The main treatment for newly discovered metastatic prostate cancer is androgen-deprivation therapy (ADT), which reduces androgen receptor activation and testicular androgen production [9]. However, responses are not durable, and tumors invariably progress to metastatic castration-resistant prostate cancer (mCRPC) [10]. Although the development of chemotherapy, anti-androgen therapy, and immunotherapy has improved overall survival for mCRPC patients, the mortality rates of patients with mCRPC remain high [11,12]. Thus, it is critical to find new therapeutic targets for the treatment of PCa, especially mCRPC.

Bloom Syndrome Protein (BLM) helicase is a member of the RecQ helicase family (RECQ1, BLM, WRN, RECQ4, RECQ5) which unwinds secondary nucleic acid structures like duplexes, Holliday junctions, G-quadruplexes, and DNA displacement loops as a 3’–5’ ATP-driven DNA helicase [13,14,15]. BLM is a crucial component of DNA damage repair (DDR) pathways, helping to maintain genomic integrity through homologous recombination repair (HRR), telomere maintenance, and replication stress reduction [16,17,18,19]. Mutations in BLM cause the autosomal recessive disease Bloom syndrome (BS) [20,21]. Multiple malignancies, including prostate, breast, and lung cancers, are more common in BS patients [22,23]. Recent studies have indicated that the BLM gene is the most important gene among seven high-risk PCa genes, and the high expression of BLM in PCa tissues is positively correlated with the malignant degree of the tumor [24]. Previous work has shown that BLM overexpression promotes PC3 cell proliferation, and knockdown of BLM promotes PCa apoptosis and inhibits PCa cell proliferation [25]. In brief, BLM is considered a potential and promising target for cancer treatment. In spite of the need for potent BLM inhibitors for further biological mechanism research or proof of concept, only a few BLM inhibitors have been reported [26,27,28,29]. Thus, it is urgent and necessary to develop novel BLM inhibitors and explore the potential anticancer strategy by targeting BLM.

In this study, we aimed to screen a novel small molecule inhibitor of BLM through virtual molecular docking from the SPECS database and put it through systematic evaluation via various experiments. The goals of this study also include confirming the inhibitory mechanisms of BLM in multiple antitumor properties, especially in DDR, and altering the p53 signaling pathway.

## 2. Results

### 2.1. BLM Is Highly Expressed in PCa and Its Expression Correlates with Poor Survival

**Figure 1:**
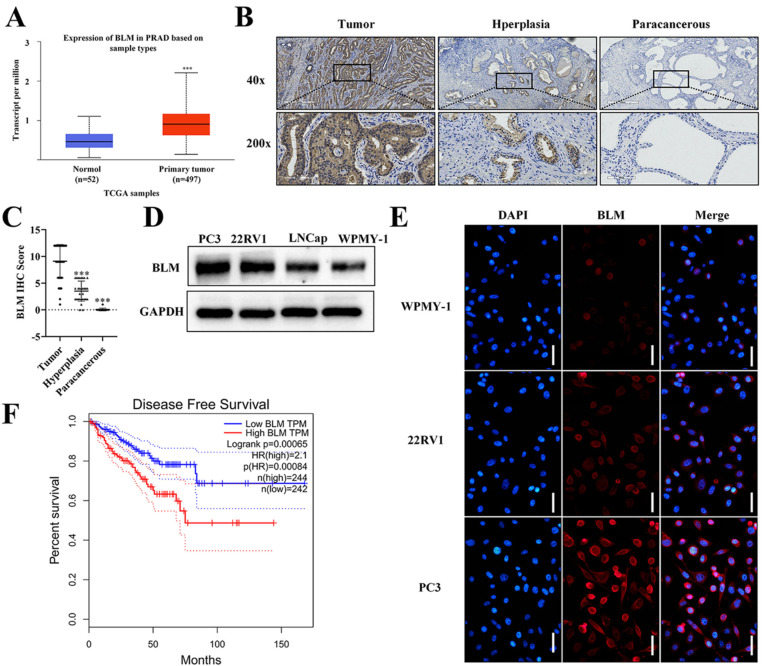
BLM is highly expressed in PCa and its expression correlates with poor survival. (**A**) Prostate cancer with BLM transcripts apparently had higher expression (n = 497) than normal tissues (n = 52) from the TCGA database (*** *p* < 0.001). (**B**) The BLM protein level in prostate cancer tissues, hyperplasia, and paracancerous tissues were shown by immunohistochemistry (IHC) (Brown: BLM). 40X and 200X-image. (**C**) BLM IHC staining scores in prostate cancer tissues (n = 60), hyperplasia tissues (n = 30), and paracancerous tissue (n = 30) are shown (*** *p* < 0.001). (**D**) WB analysis of the BLM protein level in prostate cell lines. (**E**) IF analysis of the BLM protein level in prostate cell lines. Scale bars: 50 μm. (**F**) Comparison of DFS in the indicated groups. Low BLM expression group, n = 242; high BLM expression group, n = 244. Patients with high expression of BLM had a significantly worse prognosis than those with low expression of BLM (*p* = 0.00065); n: number of patients.

The results from The Cancer Genome Atlas (TCGA) showed that the mRNA expression of BLM was significantly higher in PCa tissues than in normal tissues (Figure 1A). The results showed that BLM expression in prostate cancer was upregulated compared with that in hyperplasia and normal prostate tissue (Figure 1B,C). We further divided these patients into high (score ≥ 8) and low BLM expression groups (score < 8) and analyzed the correlation between BLM expression and several common clinicopathological features of prostate cancer (including age, clinical stage, Gleason score, Gleason grade, N-regional lymph nodes, and M-distant metastasis). We found that BLM was related to clinical stage (χ^2^ = 10.506, *p* = 0.001) and Gleason grade (χ^2^ = 9.877, *p* = 0.002) but not to age, Gleason score, N-regional lymph nodes, or M-distant metastasis *(p* > 0.05) (Table 1). To further confirm the expression of BLM in PCa, we utilized three human PCa cells (PC3, 22RV1, and LNCap) and human normal prostate cells (WPMY-1) to examine BLM protein expression by WB (Figure 1D, Supplementary Materials S1). These results confirmed that BLM was also overexpressed in PCa cells compared to normal cells. Similar results were also found via immunofluorescence analysis (Figure 1E). The results from GEPIA 2.0 analysis revealed PCa patients who had high expression of BLM showed poorer Disease-Free Survival (DFS) (*p* = 0.00065) than those with low expression of BLM (Figure 1F). In brief, these data suggest that BLM is abnormally overexpressed and predicts the poor prognosis in human prostate cancer patients. Collectively, the above results showed that BLM was strongly associated with PCa genesis and that it might act as a therapeutic drug target for PCa.

### 2.2. A High-Throughput Screen for Inhibitors of BLM

**Figure 2:**
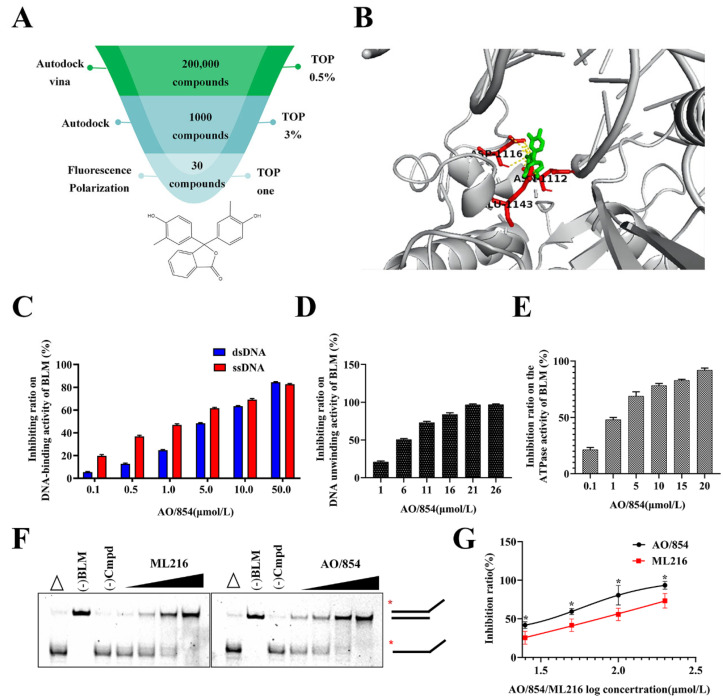
Identification of BLM inhibitors and AO/854 was an inhibitor of BLM activity in vitro. (**A**) Diagram of BLM inhibitors’ identification and structure of the hit compound (AO/854). (**B**) Docking model of the BLM-AO/854 complex. AO/854 (green) and the surrounding residues of BLM (red) were shown as sticks. (**C**) Inhibitory effects on BLM binding DNA of AO/854 by the fluorescence polarization assay. (**D**) Inhibitory effects on BLM unwinding DNA of AO/854 by the fluorescence polarization assay. (**E**) Inhibitory effects on BLM’ ATPase activity of AO/854. (**F**) Inhibitory effects on BLM unwinding DNA of AO/854 or ML216 by EMSA. In the absence of ML216 or AO/854, BLM unwinds the forked duplex into ssDNA (the third lane). The open triangle above the first lane in each case depicts heat-denatured DNA. The asterisk denotes the fluorescent end label. (**G**) AO/854 had a stronger inhibitory effect on BLM unwinding DNA than ML216 by EMSA. Data represent the average of three independent experiments. Error bars: SD (* *p* < 0.05).

By using high-throughput virtual and protein-based screening assays, we discovered an isobenzofuranone compound with the desired properties. The flow chart of our work is as follows (Figure 2A). Before the first round of docking using the Autodock Vina, an optimized database (approximately 200,000 compounds) was achieved by filtering using Lipinski’s rule of five and Veber rules and removing duplicate molecules from the Specs database (Figure S1). The top 1000 compounds from the previous step were docked with Autodock (Figure S2). The top 30 small molecules (Table S1) obtained through virtual screening were dissolved in DMSO and prepared into mother liquor at a concentration of 20 mM. We purified a truncated structure of BLM that retained helicase activity in vitro (Figure S3), selected a fluorescein-labeled dsDNA as a substrate, and used a fluorescence polarization assay to determine if the promising compounds disrupted the binding between BLM and DNA. It was found that the compound with ID number AO/854/43447428, referred to as AO/854, can significantly reduce the fluorescence polarization value of the BLM^642−1290^ and dsDNA complex (Table S2).

### 2.3. AO/854 Is a Potent Small Molecule Inhibitor of BLM

The predicted binding mode of AO/854 with BLM (PDB code 4CGZ) is shown in Figure 2B. Furthermore, the fluorescence polarization assay and EMSA were used to analyze if different concentrations of AO/854 were able to inhibit the binding and unwinding activity of the BLM^642−1290^ to dsDNA and ssDNA. Meanwhile, the reported inhibitor, ML216, was used as a reference compound. The IC_50_ values were calculated from the nonlinear regression fit. As the concentration of the AO/854 increased to 50 μM, its inhibiting ratio on dsDNA and ssDNA binding of BLM reached 84.53% and 82.76%, respectively. The results were dose-dependent (Figure 2C). AO/854 also exhibited strong inhibitory effects on the ability of BLM to unwind DNA, with IC_50_ values within 10 μM (Figure 2D) and showed better inhibitory effects than ML216 (Figure 2F,G, Supplementary Material S1). In addition, AO/854 could inhibit the ATPase activity of the BLM. As the concentration of AO/854 increased to 20 μM, its inhibiting ratio on the ATPase activity of BLM was 92.03% (Figure 2E). By adjusting the concentration of substrate ATP (0.2–1.5 mM), the effects of 10 µM and 20 µM AO/854 on the kinetic parameters of BLM^642−1290^ were determined, and then the inhibitory type of AO/854 on BLM^642−1290^ was determined. As shown in Figure S4, the double reciprocal method showed a good linear relationship, and the addition of AO/854 had no effect on Vmax, but significantly reduced the Km value, suggesting that AO/854 inhibition of BLM^642−1290^ was a competitive inhibition (Table S3). 

**Figure 3:**
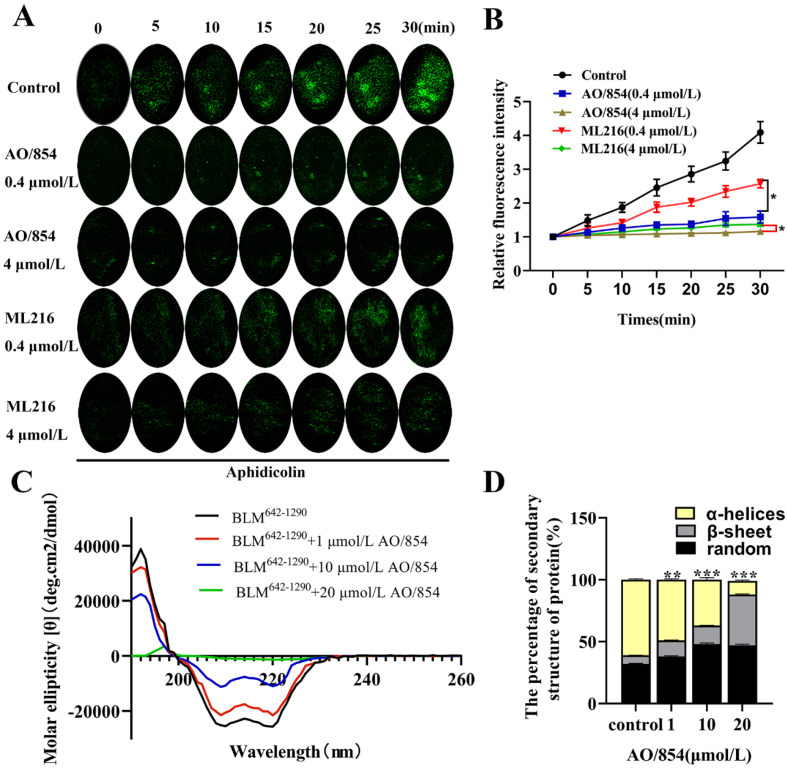
Effect of BLM inhibitors on intracellular unwinding function and BLM secondary structure. (**A**) Effect of AO/854 or ML216 on DNA helicase activity in vivo. Live cell microscopy setup for helicase activity measurements. Cells were transfected with RPA (green) as indicated 24 h before imaging. Representative confocal time-lapse microscopy images of aphidicolin-treated cells in 30 min, showing RPA accumulation from replication foci (control). After AO/854 or ML216 treatment, the accumulation of RPA was weakened. (**B**) AO/854 had a stronger inhibitory effect on DNA helicase activity in vivo. RPA accumulation at replication foci, as a proxy for helicase speed, was measured for each time point, normalized to the GFP channel values at time point 0 (pretreatment), and the coefficient of variation of the GFP-channel intensities was plotted for each time point. Data represent the average of three independent experiments. Error bars: SD (* *p* < 0.05). (**C**) CD spectra of BLM and their changes after binding to different concentrations of AO/854. (**D**) Protein secondary structure contents of AO/854 (1, 10, and 20 μmol/L)-treated BLM^642−1290^ groups compared with DMSO-treated group (control group). Data represent the average of three independent experiments. Error bars: SD. (** *p* < 0.01, *** *p* < 0.001).

To test the influence of the inhibitor of BLM on the helicase unwinding activity in vivo, we designed a method for measuring intracellular helicase rate according to the reports of Rausch et al. [30]. In brief, we used C2C12 mouse myoblasts to instantaneously transfect GFP-labeled RPA34 protein expression vector and determined the helicase unwinding rate through accumulation of RPA. When the activity of the helicase was higher and the speed of the helicase was faster, the accumulation of RPA was higher, and the fluorescence intensity was stronger. When the activity of helicase was inhibited, the accumulation of RPA decreased, and the fluorescence intensity weakened. The results showed that the accumulation of RPA in the nucleus was time-dependent under the action of the aphidicolin. In the cells treated with AO/854 or ML216, the cumulative amount of RPA decreased, indicating that the above two compounds have a certain inhibitory effect on the helicase function of cells in a dose-dependent manner. The results also showed that AO/854 had a stronger inhibitory effect on the dissociation function of C2C12 cells than ML216 (Figure 3A,B). Next, a CD assay was used to detect if AO/854 influenced the secondary structure of the BLM^642−1290^ helicase (Figure 3C,D). The chromatogram of the BLM^642−1290^ was a typical chromatogram of an α-helix structure, which included a positive absorption spectrum band around 192 nm and two negative absorption spectra bands around 208 and 222 nm. The peaks at 192, 208, and 222 nm showed a decreasing concentration-dependent trend after AO/854 was mixed with the BLM^642−1290^. The ratio of α-helix was analyzed by DICRO2000 software. The results showed that the α-helix ratio of the BLM^642−1290^ was 61%, which decreased to 49% after being mixed with 1 μM AO/854. As the concentration of AO/854 increased to 10 μM and 20 μM, the α-helix ratio changed to 37% and 11%, respectively. This result indicated that the α-helix of BLM^642−1290^ was gradually destroyed by an increasing concentration of AO/854. In conclusion, we selected AO/854 as an inhibitor of BLM from the Specs database for further study.

### 2.4. AO/854 Represses Cell Proliferation and Migration in PCa Cells

**Figure 4:**
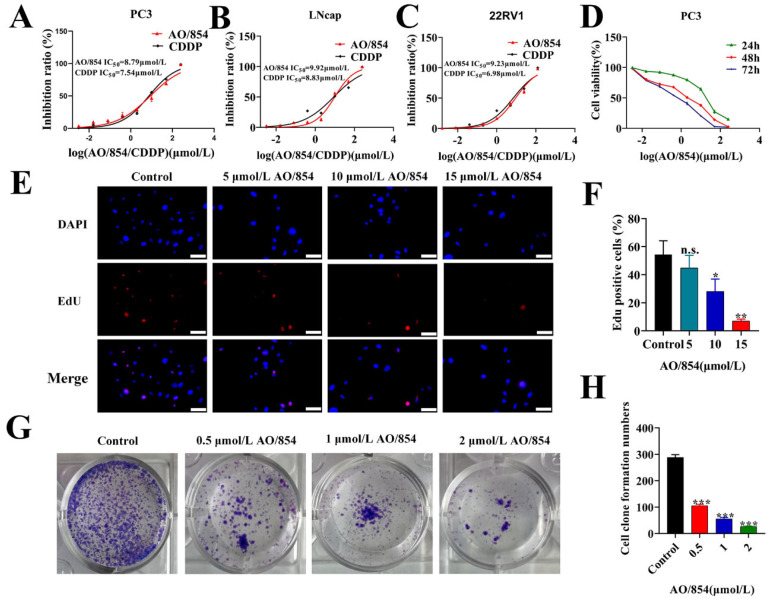
AO/854 repressed proliferation in prostate cancer cells. (**A**–**C**) Inhibition ratios in prostate cancer cells following 48 h treatment: for PC3 cells, AO/854 IC_50_—8.79 μmol/L and CDDP IC_50_—7.54 μmol/L (**A**); for LNcap cells, AO/854 IC_50_—9.92 μmol/L and CDDP IC_50_—8.83 μmol/L (**B**); and for 22RV1 cells, AO/854 IC_50_—9.23 μmol/L and CDDP IC_50_—6.98 μmol/L (**C**). (**D**) Inhibitory effects of AO/854 on cell viability of PC3 cells. (**E**) Inhibitory effects of AO/854 on the proliferative activity of PC3 cells by the EdU assay. Scale bars: 50 μm. (**F**) Quantification of EdU experiments in AO/854 (5, 10, 15 μmol/L)-treated groups compared with DMSO-treated group (control group). Data represent the average of three independent experiments. Error bars: SD. (n.s. *p* > 0.05, * *p* < 0.05, ** *p* < 0.01). (**G**) Inhibitory effects of AO/854 on colony formation activity of PC3 cells by colony formation assay. PC3 cells were treated with AO/854 at concentrations of 0.5, 1, and 2 μmol/L for about 1 week. (**H**) Quantification of colony formation experiments in AO/854 (0.5, 1, 2 μmol/L)-treated groups compared with DMSO-treated group (control group). Data represent the average of three independent experiments. Error bars: SD. (*** *p* < 0.001).

In order to investigate the repressive effects of AO/854 on PCa cells, PC3, 22RV1, and LNCap cells were treated with AO/854 via a CCK8 assay. CDDP was used in these experiments as a positive control. As shown in Figure 4A–C, the results showed that AO/854 possessed strong proliferation inhibition against 22RV1, LNCap, and PC3. After 48 h of treatment, the IC_50_ values of BLM and CDDP against PC3, LNCap, and 22RV1 were 8.79 and 7.54 μM, 9.92 and 8.83 μM, and 9.23 and 6.98 μM, respectively. We also tested the efficacy of ML216 on the 22RV1, LNCap, and PC3 cells and determined that the IC_50_ values were higher than 50 μM. AO/854 has a stronger proliferation inhibition against 22RV1, LNCap, and PC3 cells compared with ML216. A CCK8 assay was used to determine the viability of PC3 cells after treatment with AO/854 for different times. The results showed that the growth of PC3 cells exposed to AO/854 was inhibited in a time-related and concentration-related manner (Figure 4D). Similar results were also found via Edu analysis (Figure 4E,F). At a concentration of 0.5 μM, AO/854 significantly reduced the colony formation of PC3 cells (around 65% viable). When the concentration was increased to 2 μM, colony formation was completely ablated in PC3 cells (Figure 4G,H). 

**Figure 5:**
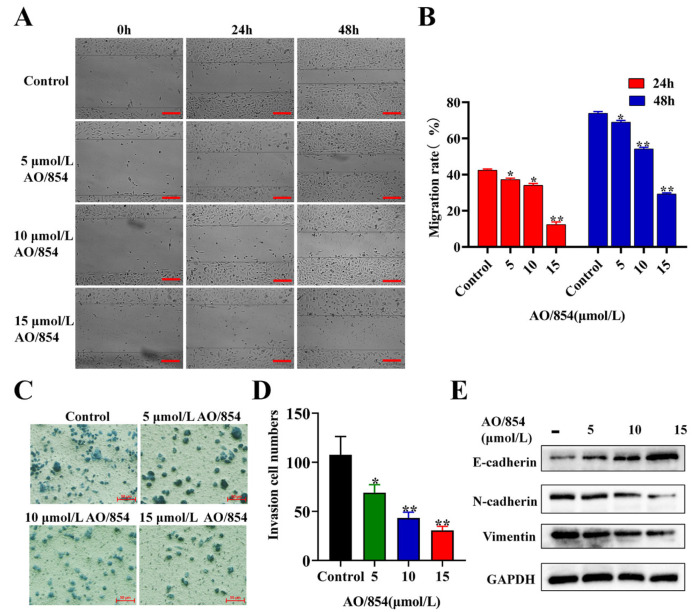
AO/854 repressed cell invasion and migration in PC3 cells. (**A**) Inhibitory effects of AO/854 (5, 10, and 15 μmol/L) on the migration activity of PC3 cells by scratch wound-healing assay. Scale bars: 50 μm. (**B**) Quantification of migration experiments in AO/854 (5, 10, and 15 μmol/L)-treated groups compared with DMSO-treated group (control group). (* *p* < 0.05, ** *p* < 0.01). (**C**) Inhibitory effects of AO/854 (5, 10, and 15 μmol/L) on invasion activity of PC3 cells by transwell assay. Scale bars: 50 μm. (**D**) Quantification of invasion experiments in AO/854 (5, 10, and 15 μmol/L)-treated groups compared with DMSO-treated groups (control group). (* *p* < 0.05, ** *p* < 0.01). (**E**) Protein levels of E-cadherin, N-cadherin, and vimentin, as detected by western blotting after being treated with AO/854 (5, 10, and 15 μmol/L) for 48 h. GAPDH was examined as a loading control.

To know whether AO/854 influences the metastasis of PC3 cells, the migration assay was performed by the wound-healing assay and transwell assay. In the wound-healing assay, after incubation with DMSO or AO/854 (5, 10, and 15 μM) for 24 or 48 h, the migration area was significantly larger in cultures with a treatment of AO/854 (Figure 5A,B). In the transwell assay, at a concentration of 5 μM, AO/854 significantly reduced the invasion numbers of PC3 cells (around 36% viable) (Figure 5C,D). According to the results, we used three biomarkers (E-cadherin, N-cadherin, and vimentin) to evaluate the migration status of PC3 cells. The results showed a significant decrease in N-cadherin, vimentin, and an increase in E-cadherin (Figure 5E, Supplementary Materials S1). These experiments suggest that the migration ability was induced by AO/854 treatment in a dose-dependent manner in PC3 cells.

### 2.5. Flow Cytometry Cell Cycle Arrest and Apoptosis Analysis of AO/854

**Figure 6:**
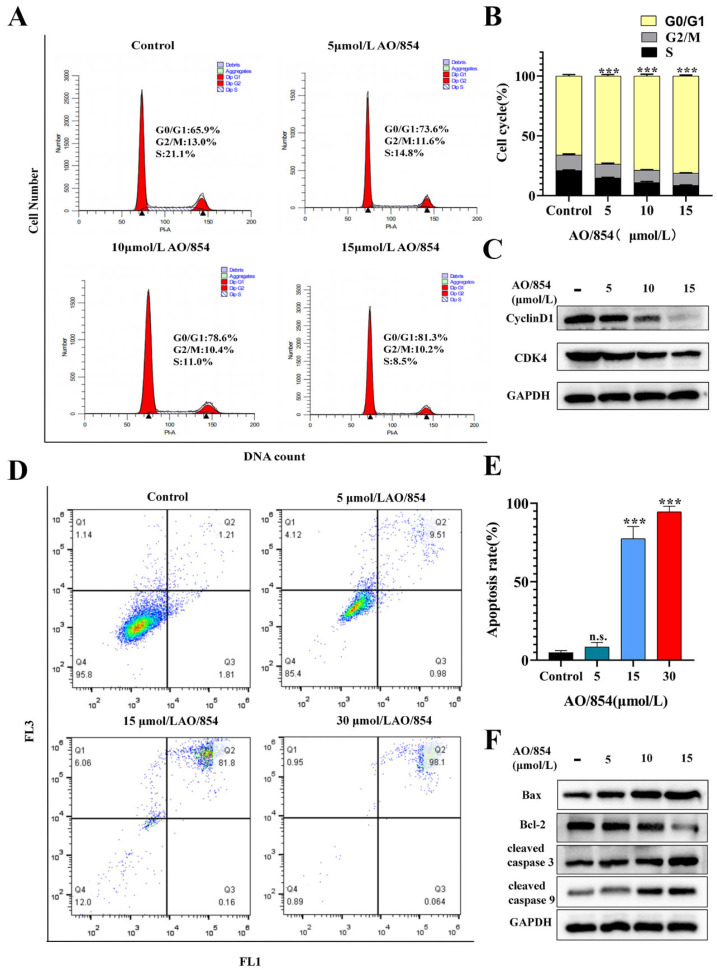
AO/854-induced cell cycle arrest and cell apoptosis in PC3 cells. (**A**) Cell cycle analysis for control, AO/854 (5, 10, and 15 μmol/L) treatment on PC3 cells. (**B**) Quantification of cell cycle experiments in AO/854 (5, 10, and 15 μmol/L)-treated groups compared with DMSO-treated group (control group). (*** *p* < 0.001). (**C**) Protein levels of CDK4 and CyclinD1 as detected by western blotting after being treated with AO/854 (5, 10, and 15 μmol/L) for 48 h. GAPDH was examined as a loading control. (**D**) Cell apoptosis analysis for control, AO/854 (5, 15, and 30 μmol/L) treatment on PC3 cells. (**E**) Quantification of cell apoptosis experiments in AO/854 (5, 10, and 15 μmol/L)-treated groups compared with DMSO-treated group (control group) (n.s. *p* > 0.05, *** *p* < 0.001). (**F**) Protein levels of Bax, Bcl-2, cleaved caspase 3, and cleaved caspase 9 as detected by western blotting after being treated with AO/854 (5, 10, and 15 μmol/L) for 48 h. GAPDH was examined as a loading control.

The BLM is a crucial component of HRR pathways, helping to maintain genomic integrity, and the deficiency of BLM might induce cell cycle arrest and apoptosis. To test the influence of AO/854 on cell cycle arrest and apoptosis, PC3 cells were treated with AO/854 via a flow cytometry assay. As a result of cell cycle arrest, the respective percent arrest (G0/G1 phases) of AO/854 (5, 10, and 15 μM) on PC3 cells (73.6, 78.6, and 81.6%) was higher than untreated cell lines (65.9%) (Figure 6A,B). The expression of cell cycle-related proteins could be changed by AO/854. Compared with the control group, the expression levels of the CDK4 and CyclinD1 proteins were downregulated in the AO/854 groups (Figure 6C, Supplementary Materials S1). The PC3 cells treated with DMSO (control) had 95.80% viability, whereas the apoptotic percent with AO/854 (5, 10, and 15 μM) treatment was 10.49%, 81.96%, and 98.16%, respectively (Figure 6D,E). To further evaluate the effects of AO/854 in PC3 cells, the levels of apoptosis-related proteins were determined. The expression of proteins showed a significant decrease in Bcl-2 and an increase in Bax, cleaved-caspase3, and cleaved-caspase9 (Figure 6F, Supplementary Materials S1). The experiments were performed in triplicate and similar results were obtained. 

### 2.6. DNA Damage Is Observed in PC3 Cells Surviving AO/854 Treatment

**Figure 7:**
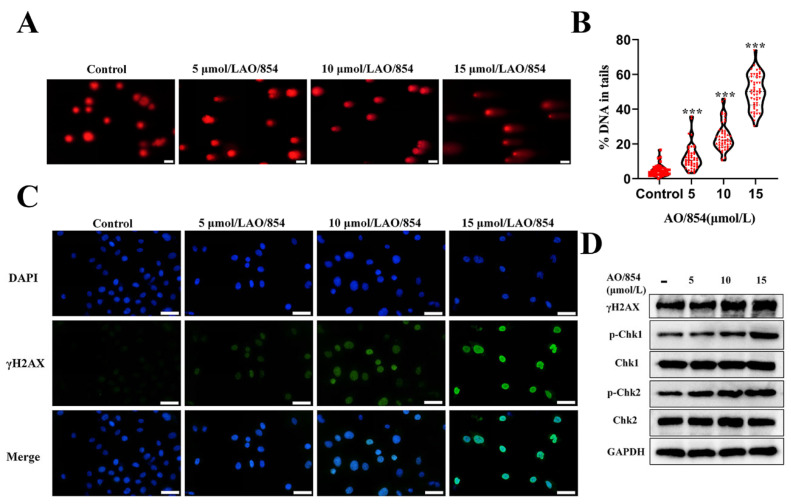
AO/854-induced DNA damage in PC3 cells. (**A**) Comet assay of PC3 cells treated with AO/854 (5, 10, and 15 μmol/L) for 48 h. Scale bars: 50 μm. (**B**) Quantification of %DNA in tails in AO/854 (5, 10, and 15 μmol/L)-treated groups compared with DMSO-treated group (control group). Quantification of 5 fields with 100 cells in total (*** *p* < 0.001). (**C**) Detection of γH2AX foci (green) 48 h after AO/854 (5, 10, and 15 μmol/L) treatment. Scale bars: 50 μm. (**D**) Protein levels of γH2AX, p-Chk1, Chk1, p-Chk2, and Chk2 as detected by western blotting after treated with AO/854 (5, 10, and 15 μmol/L) for 48 h. GAPDH was examined as a loading control.

BLM was an important DNA repair factor in the HRR pathway, and the deficiency of BLM could induce DDR. To examine whether treatment of PC3 cells with AO/854 results in DNA damage and subsequent activation of DDR pathways, we began by using PC3 cells responsive to AO/854 therapies. Firstly, a comet assay directly detected the DNA damage after a 48 h AO/854 treatment, as evidenced by an increased DNA in tails. The results revealed that AO/854 caused a significant increase in DNA strand breaks in a dose-dependent manner in PC3 cells (Figure 7A,B). We then determined the γH2AX expression (a DNA damage marker) on PC3 cells upon treatment of AO/854 by the IF assay. We observed remarkable increased γH2AX foci (green) in PC3 cells treated with AO/854(Figure 7C). We evaluated its effect on the DNA damage pathway because AO/854 has a significant impact on the DNA damage. As shown in Figure 7D and Supplementary Materials S1, the level of major damage sensors in damage response, such as p-Chk1 and p-Chk2, was obviously upregulated with the treatment of AO/854. Taken together, the BLM inhibitor AO/854 could significantly activate DNA damage pathways.

### 2.7. Identification and Bioinformatics Analysis of Differently Expressed Proteins (DEPs) in PC3 Cells after AO/854 Treatment

**Figure 8:**
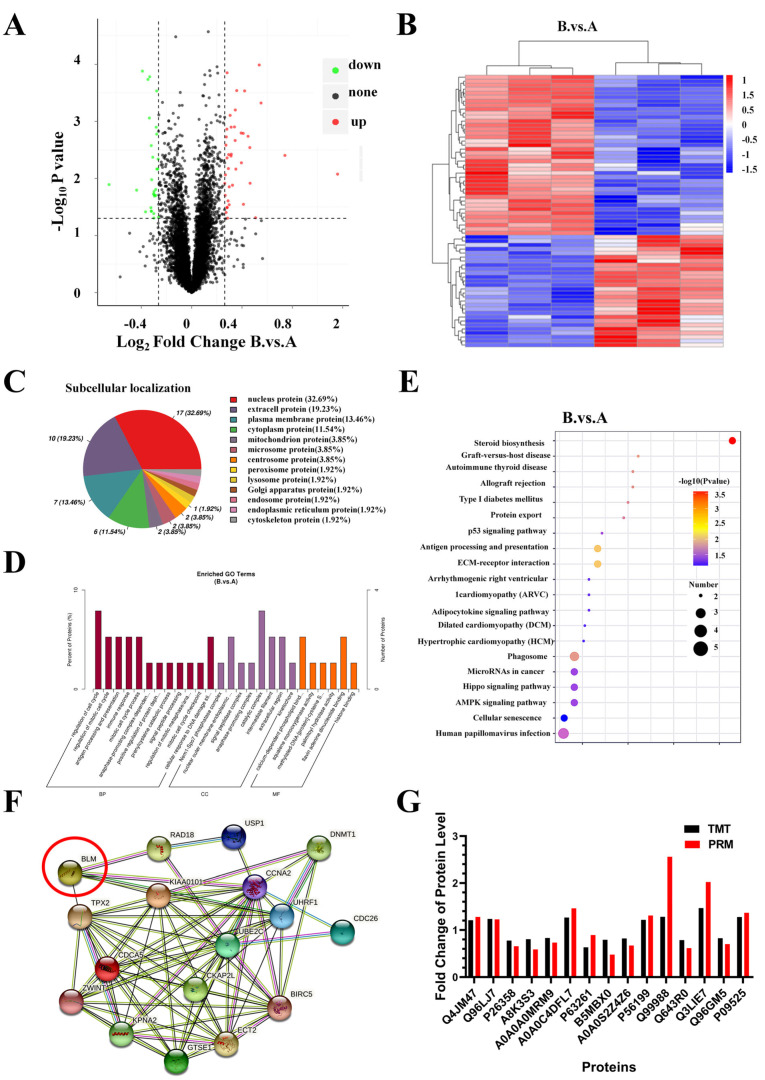
Identification and bioinformatics analysis of differently expressed proteins (DEPs) in PC3 cells after AO/854 treatment. (**A**) Volcanic map of DEPs in PC3 cells after AO/854 (5 μmol/L) treatment. (**B**) Cluster heat map of DEPs in PC3 cells after AO/854 (5 μmol/L) treatment. (**C**) Subcellular localization analysis of DEPs in PC3 cells after AO/854 (5 μmol/L) treatment. (**D**) Gene ontology (GO) analysis of DEPs in PC3 cells after AO/854 (5 μmol/L) treatment. (**E**) KEGG analysis of DEPs in PC3 cells after AO/854 (5 μmol/L) treatment. (**F**) Protein–protein interaction networks for DEPs and BLM. (**G**) Confirmation of 15 selected differently produced proteins detected by PRM technique. Note: Group A is the negative control group; Group B is the AO/854 treatment group (5 μmol/L).

To explore the underlying mechanism through which AO/854 inhibits cell proliferation and induces cell apoptosis in PCa cells, we next performed TMT-based quantitative proteomics analysis to identify the proteins that were regulated by AO/854 treatment in PC3 cells. The AO/854-treated group (group B) showed a significantly different proteome expression profile than the control group (group A), with 40 DEPs upregulated and 28 DEPs downregulated (Figure 8A,B, Table S4), as demonstrated by the volcano plots and hierarchical clustering analysis (Student’s *t*-test, *p* < 0.05, fold change [FC] > 1.2 or <0.83). A proteomics study of subcellular localization revealed that after AO/854 treatment, approximately 32.69% of DEPs were localized in the nucleus, implying that AO/854 primarily targets nuclear proteins (Figure 8C). To learn more about the roles of the DEPs generated by AO/854, researchers used GO annotation enrichment analysis and the KEGG pathway enrichment analysis. GO annotation analysis showed that the DEPs induced by AO/854 were mostly enriched in 12 biological processes, 8 cell components, and 6 molecular functions (Figure 8D). Figure 8E depicts the top ten most enriched and KEGG pathways. It is worth noting that the ‘p53 signaling pathway’ was one of the top three KEGG pathways with the most significant enrichment. The BLM-DEPs interaction network was constructed using the STRING database. There was a close interaction between DEPs and BLM (Figure 8F). Because PRM is a widely used and effective method for precisely quantifying and verifying an array of target proteins of interest, it was employed to further validate the label-free quantitative proteomic finding. For PRM analysis in this study, fifteen differently generated proteins involved in these GO keywords and pathways were chosen for analysis (Table S5). Furthermore, for each protein, two distinct peptides with expected chemical stability were chosen, and relative protein abundance was calculated as the average of the two normalized peptide peak regions (Figure S5). Figure 8G shows the PRM result, which showed the same trends as the label-free quantitative proteomics data.

**Figure 9:**
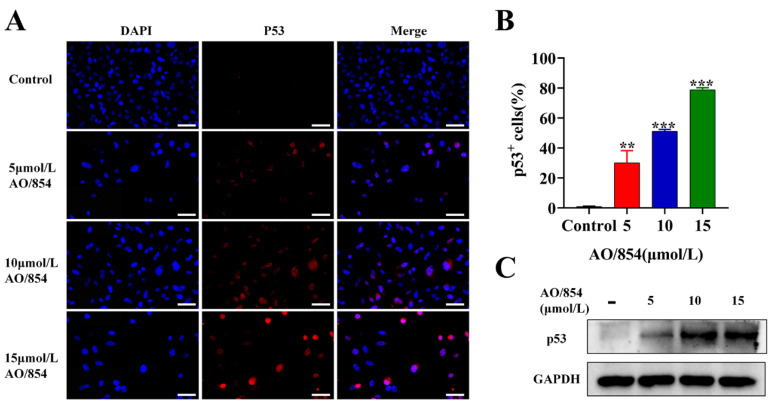
AO/854 reactivated or restored p53 signal pathway in PC3 cells. (**A**) Detection of p53 foci (red) 48 h after AO/854 (5, 10, and 15 μmol/L) treatment. Scale bars: 50 μm. (**B**) Quantification of p53^+^ cells in AO/854 (5, 10, and 15 μmol/L)-treated groups compared with DMSO-treated group (control group). Quantification of 5 fields with 100 cells in total. (** *p* < 0.01, *** *p* < 0.001). (**C**) Protein levels of p53 as detected by western blotting after being treated with AO/854 (5, 10, and 15 μmol/L) for 48 h. GAPDH was examined as a loading control.

In order to investigate the effects of AO/854 on the p53 pathway in PC3 cells, we then determined the p53 expression on PC3 cells upon treatment of AO/854 (5, 10, and 15 μM) by the IF and WB assays. The results of IF revealed that PC3 cells are p53-null and that after AO/854 treatment, the expression of p53 protein was upregulated (Figure 9A,B). Similar results were also found via WB (Figure 9C, Supplementary Materials S1). Taken together, these data demonstrate that AO/854 as an inhibitor of BLM can induce DNA damage, activate DNA damage pathways, and then reactivate or restore the p53 signaling pathway, resulting in cell G0/G1 arrest, cell apoptosis, and inhibition of cell metastasis.

### 2.8. AO/854-Suppressed Tumour Growth in PC3 Xenograft Model

**Figure 10:**
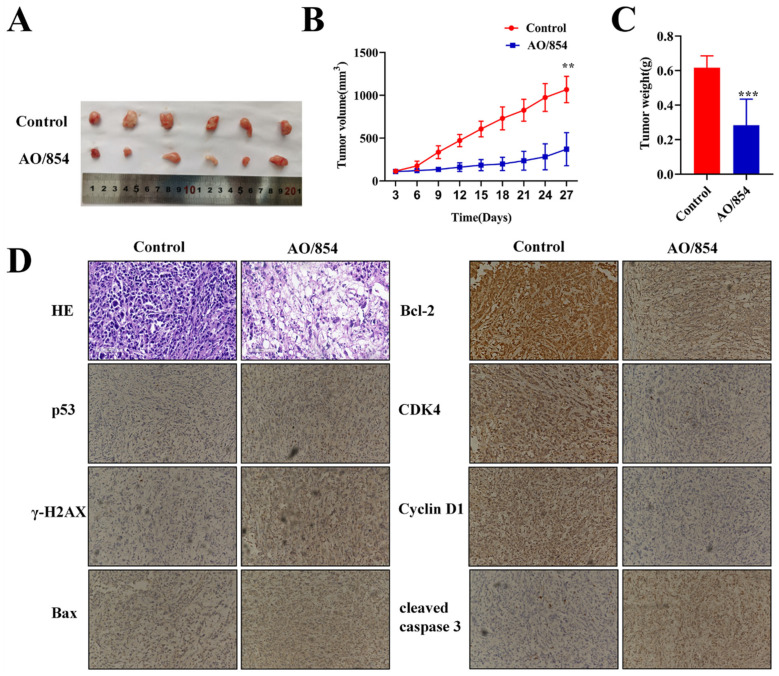
AO/854-suppressed tumor growth in the PC3 xenograft model (n = 6). (**A**) Representative images of PC3 xenograft tumors after AO/854 (2 mg/kg, once every two days) or 0.9% saline treatment. (**B**) Tumor growth curves in PC3 xenograft models. (** *p* < 0.01). (**C**) Quantification of tumor weight in AO/854 (2 mg/kg, once every two days)-treated group compared with the 0.9% saline-treated group (control group). (*** *p* < 0.001). (**D**) Representative images of HE staining and immunohistochemical staining analyses of p53, γH2AX, Bax, CDK4, CyclinD1, and cleaved caspase 3 in tumor tissues.

In vivo drug treatment experiments using male BALB/c mice were subsequently performed to confirm the validity of the above results. Twelve nude mice were modeled, and the tumor formation rate was 100%. No accidental deaths occurred during feeding. When the tumor volume was approximately 100 mm^3^, the mice were randomly assigned and given intraperitoneal administration as planned. The tumor weight and volume were relevantly regressed after treatment of AO/854 (2 mg/kg, once every two days). On the 27th day, the inhibition rate of tumor volume was as high as 64.34%, as shown in Figure 10A,B. The average weight of tumor masses in the control group was 0.62 g, and that of the AO/854-treated group was 0.28g (Figure 10C). HE staining was used to observe the antitumor effect of AO/854 at the histological level. The results showed that the tumor tissue of the control group had a dense, complete nucleus, and the nucleus was big and deeply dyed. After treatment, the number of tumor cells decreased significantly compared with before and the arrangement was relatively loose, which indicated that the tumor cells were killed by AO/854 and the cells showed apoptosis. Next, an IHC assay was carried out with the tumor tissues. The results showed that the treatment of AO/854 decreased the protein levels of CyclinD1, CDK4, and Bcl-2 and increased the protein levels of p53, cleaved-caspase 3, and γH2AX in tumors. In general, the results were consistent in vivo and in vitro (Figure 10D).

## 3. Discussion

BLM is a DNA helicase that performs important roles in DDR pathways and is considered an attractive target for cancer therapy. In this study, we reported the discovery of a novel inhibitor of BLM, named AO/854, based on virtual molecular docking, and put it to systematic evaluation via various experiments. 

According to recent research, BLM levels are elevated in a variety of cancers, including lung squamous cell carcinoma, colon adenocarcinoma, endometrial carcinoma, cervical squamous cell carcinoma, and endocervical adenocarcinoma [31,32]. Additionally, patients with lung cancer and stomach cancer have inferior overall survival rates when BLM is overexpressed [33]. Recently, clinicopathological analysis confirmed that BLM was the most important gene among seven high-risk PCa genes. BLM overexpression promotes PC3 cell proliferation, and knockdown of BLM promotes PCa apoptosis and inhibits PCa cell proliferation [24,25]. In agreement with these findings, our research showed that BLM is highly expressed in prostate cancer tissue and cell lines, and the high expression of BLM in PCa tissues is positively correlated with the malignant degree of the tumor. The overexpression of BLM is also associated with worse survival in PCa. Therefore, BLM might be considered an attractive target for PCa therapy. ML216 is the first BLM helicase inhibitor. Later, Yin, Wang, and Zhang successively found some other BLM inhibitors. However, only a few inhibitors have been proven to have anticancer properties. Screening for new BLM inhibitors is a possible direction for the treatment of PCa. To accomplish this goal, we screened the Speces databases using high-throughput virtual and fluorescence polarization assays. A compound designated AO/854 appeared to be specific based on its reduced ability to affect DNA binding to BLM. BLM interacted with DNA mainly through residues 1112, 1125 and other amino acids in the RQC structure domain [34]. Here, in our docking model of BLM-DNA in a complex with AO/854, AO/854 was suitable for the DNA binding pocket in the RQC domain of BLM, and residues 1112, 1116, and 1143 amino acids were adjacent to the allosteric pocket where AO/854 is located. A panel of in vitro assays, including fluorescence polarization assay, EMSA, and ATPase activity analysis assay, were performed to support that AO/854 could disrupt the interaction of BLM and DNA, affect unwinding activities, and ATPase activity of BLM in vitro. After that, we identified that compound AO/854 possessed the best inhibitory effect on BLM for further study.

During the cellular analyses of the effects of AO/854, we analyzed whether this compound, through targeting BLM, was implicated in cell survival, proliferation, apoptosis, as well as migration cell function. The results revealed that a more significant proliferation arrest appeared in a dose-dependent manner of AO/854 than ML216 in 22RV1, LNCap, and PC3 cells. In addition, we also discovered that AO/854, an inhibitor of BLM, reduced cell migration and invasion in PC3 cells. In order to further explore the mechanism, subsequent biological assays, cell cycle and apoptosis analysis, alkaline comet assay, proteome and WB, were applied and analyzed to determine that AO/854, an inhibitor of BLM, might activate cell-cycle checkpoint responses, DNA damage responses, or other pathways. AO/854 (5, 10, and 15 μM) treatment of PC3 cells for 48 h caused cell-cycle arrest in G0/G1, a decrease in the number of cells in S-phase, and downregulation of CDK4 and CyclinD1 proteins. Other researchers support our findings. Recent research showed that BLM depletion has been reported to suppress cell proliferation in human fibroblasts, and BLM-deficient cells have defective S-phase progression. In another study, knockdown of BLM in PC3 cells suppressed cell proliferation, decreased the number of cells in S-phase, and increased cell S-phase arrest [25]. BLM participates in the regulation of cyclin-dependent protein serine/threonine kinase activity, which is why BLM is expressed in PC cells as a ‘regulatory mechanism for protein phosphorylation’ [35]. In the cell cycle, BLM expression is lowest in the G1 phase, significantly higher in the S phase, and highest in the G2/M phase [36,37]. BLM is phosphorylated at Ser144 and interacts with the monopolar spindle 1 spindle assembly checkpoint kinase. In order to maintain genomic stability in both healthy and tumor tissues, phosphorylated BLM may subsequently interact with Polo-like kinase 1 via its polo-box domain [38,39,40,41,42]. Together, these data highlight the inhibition of BLM can activate cell-cycle checkpoint responses.

DDR pathways are encoded by a class of proteins that detect DNA double-strand breaks, chromosomal fragmentation, translocations, and deletions, and can correct some alterations. Several germline mutations of DDR genes, including BRCA2 (13% of cases), ATM (7.3%), MSH2 (2%), and BRCA1 (0.3%), were discovered by the Cancer Genome Atlas Research Network in 333 individuals with primary PCa. To further clarify the significance of these mutations in PCa, it has been estimated that up to 20–25% of mCRPC patients had mutations in the DDR gene pathways [43]. At present, BLM is considered to play a crucial role in the DDR pathway in cells. BLM is recruited to DNA replication forks after replicative stress, which is generated by DNA damage or replication arrest. BLM interacts with TopIII α, RMI1, RMI2, FANCM, and FANCC, bridging key components required for proper DNA repair to dissolve the double Holiday Junctions, an intermediate formed during HRR after the resection step [44]. This results in the dissolution of the double Holiday Junction, which prevents genetic crossover. Inhibition of BLM may affect DNA stability and the DDR pathway. ML216, an inhibitor of BLM, modulates the role of BLM in genome maintenance in human cells and increases the frequency of SCEs [45]. Another inhibitor of BLM compound 9h could effectively disrupt BLM recruitment to DNA in cells and trigger DDR at the telomere region [27]. A group of new BLM inhibitors known as isaindigotone derivatives were shown to interfere with BLM/DNA interactions and control HRR by encouraging the accumulation of Rad51 in cancer cells [28]. Similar to the above results, our results showed that BLM inhibition by AO/854-induced DNA damage, suggesting that inhibition of BLM may break the balance of DNA repair. Additionally, the increased p-Chk1 and p-Chk2 by AO/854 in PC3 cells suggested that AO/854 activated the DDR pathways. Similar results were obtained with other BLM inhibitors.

The tumor suppressor gene, *p53*, is a key cellular component in maintaining genomic stability. Arguably, the prime p53-dependent tumor suppressive response is the induction of apoptosis, which is the goal of many anti-cancer therapies that are aimed at the reactivation or restoration of wild-type p53 function [46]. Both BLM and p53 are key factors in DDR. BLM has been shown to bind to the dephosphorylated C-terminal domain of p53, and it has been suggested that this binding may modulate the induction of p53-mediated apoptosis and possibly DNA repair [47]. Ectopic expression of the fragment of wild-type BLM containing the p53-interactive domain suppresses p53-mediated transcription and interferes with p53-mediated growth inhibition. Bloom syndrome cells undergo p53-dependent apoptosis and delayed assembly of BRCA1 and NBS1 repair complexes at stalled replication forks. In a previous study, the BLM/EZH2/MDM2/p53 axis, which is associated with cell proliferation and metastasis, was found to be associated with the p53 signaling pathway in PC3 cells [48]. In this study, the proteomics analysis results showed that BLM inhibition by AO/854 in PC3 cells changed many protein expressions, which had a close interaction with BLM, and the ‘p53 signaling pathway’ was one of the top three KEGG pathways with the most significant enrichment. In PC3 cells, an exon 5 single base deletion was found that created a stop signal in exon 5. The absence of detectable p53 RNA in PC3 cells rendered them incapable of producing any p53 protein, robbing wild-type p53 of its function [49]. In our study, the results of WB and IF revealed that PC3 cells are p53-null, and that after AO/854 treatment, the expression of p53 protein was upregulated. The AO/854 may reactivate or restore the wild-type p53 function of PC3 cells. AO/854-induced cell apoptosis by upregulating modulators of apoptosis (p53, Bax) and inhibiting modulators of anti-apoptotic (Bcl-2) in PC3 cells. Our study has indicated that AO/854, an inhibitor of BLM, elicits DNA damage and triggers the DDR and downstream p53-dependent apoptosis in PC3 cells.

Such studies, although providing valuable information, have some limitations. These studies had relatively small sample sizes of patient tissues, and the results need to be further studied and verified. There is still relatively limited data mining and utilization in proteomic research, and the differential genes and pathways discovered will be studied in-depth in the future.

## 4. Materials and Methods

### 4.1. Patient Samples

In this study, 60 PCa and 30 matched paracancerous tissue samples and 30 hyperplasia tissue samples were obtained from the Zunyi Medical University (Zunyi, China) between 2017 and 2019. All samples were obtained from radical prostatectomy. The inclusion criteria were as follows: The patient was diagnosed with prostate cancer by transrectal prostate biopsy guided by preoperative ultrasound, and patients within 1 month after radical prostatectomy have low prostate-specific antigen (PSA) concentrations (<0.1 ng/mL). The exclusion criteria were as follows: (1) Patients with incomplete clinical and pathological data; (2) Previous prostate surgery history; (3) Preoperative patients receiving neoadjuvant therapy such as chemotherapy or endocrine therapy; (4) Postoperative patients receiving adjuvant radiotherapy or endocrine therapy. The processes used to obtain tissue samples followed strict scientific methods and were approved by the Ethics Committee of the Zunyi Medical University.

### 4.2. Cell Culture and Animals

A human prostatic stromal myofibroblast cell line (WPMY-1) and prostate cancer cell lines (PC3, 22RV1, and LNCap) were purchased from the Zhong Qiao Xin Zhou Biotechnology Co., Ltd. (Shanghai, China). PC3, LNCap, and WPMY-1 cells were cultured in DMEM/F12 medium (Gibco, Grand Island, NY, USA). 22RV1 cells were cultured in RPMI 1640 (Gibco). All culture media were supplemented with 10% FBS (04-001-1ACS; BI, Beit HaEmek, Israel), 1% penicillin (100 U/mL), and streptomycin (100 g/mL) solution (SV30010; HyClone, Logan, UT, USA).

BALB/c Nude mice (4 to 6 weeks old) were purchased from Hua Fu Kang Biotechnology Company (Beijing, China) and were reared according to the Laboratory Animal Welfare Ethics Committee of Zunyi Medical University.

### 4.3. Plasmids, Antibodies and Reagents

GFP-tagged human RPA34 was created by Tsingke Biotechnology Co., Ltd. (Beijing, China) and is used to study replication protein A (RPA) accumulation.

pET-15b-BLM^642−1290^ BL21 recombinant *E. coli* Condon Plus was a gift from Dr. Xuguang Xi of Northwest A & F University.

Primary antibodies: BLM(bs-12872R, Biosynthesis Biotechnology, Beijing, China); Bcl-2 (ab182858, Abcam, Cambridge, UK); cleaved caspase 3 (ab2302, Abcam); cleaved caspase 9 (9505, Cell Signaling Technology, Danvers, MA, USA); E-cadherin (PB9561, Boster Biological Technology, Pleasanton, CA, USA); N-cadherin (PA1328, Boster Biological Technology); vinmentin (PB9359, Boster Biological Technology); CDK4 (11026-1-AP, Proteintech, Wuhan, China); CyclinD1 (60186-1-Ig, Proteintech); BAX (50599-2-Ig, Proteintech); p53 (60283-2-Ig, Proteintech); Chk1 (380200, Zen BioScience, Chengdu, China); Phospho-chk1 (Ser280) (310037, Zen BioScience); Chk2(R23921, Zen BioScience); Phospho-Chk2 (Thr68) (340766, Zen BioScience); GAPDH (60004-1-Ig, Proteintech).

Positive control cisplatin (CDDP) and ML216 were purchased from Topscience (Shanghai, China). Thirty small-molecule compounds were obtained from the SPECS database (Topscience, Shanghai, China).

Sangon Biotech (Shanghai, China) synthesized a 45-nt single-stranded DNA (ssDNA, A1: 5′-AATCCGT CGAGCAGTTTAGGTTAG GTTAGTTTTTT-3′) and a fluorescein-labeled 21-nt single-stranded DNA (ssDNA, A2: 3′-FAM-TTAGGCAGC TCGTCTCAATCC-5′). Two complementary ssDNAs were 1:1 mixed in buffer (20 mM Tris, 100 mM NaCl, pH 7.9), warmed at 85 °C for 5 min, and cooled to 20 °C. The double-stranded DNA (dsDNA, A1A2) was used to detect DNA binding or unwinding activity of BLM.

### 4.4. Bioinformatics Analysis

The GEPIA (http://gepia2.cancer-pku.cn/#index, accessed on 15 May 2021) is a freely available tool that incorporates information from the Genotype Tissue Expression (GTEx) and Cancer-Genome Atlas (TCGA) projects for logistic regression and genetic association analysis of patient outcomes. The BLM expression in PCa was compared with the normal cases. In addition, the Kaplan–Meier curve of disease-free survival according to the various levels of BLM was assessed.

### 4.5. Immunohistochemical (IHC) Staining and Immunofluorescence (IF) Analysis

All clinical patient tissue paraffin slides were autoclaved in 10 mM citric sodium (pH 6.0) for 15 min to unmask antigens after being treated with 3% H_2_O_2_ for 10 min. After being rinsed in phosphate-buffered saline three times, the tissue slides were incubated with primary BLM antibodies (1:100) at 4°C overnight, followed by incubation with secondary antibodies for one hour at 37°C. The intensity score was evaluated by multiplying the percentage of positive cells (0 = none; 1 = 1–10%; 2 = 11–50%; 3 = 51–80%; 4 = 81–100%) by the intensity (0 = none; 1 = weak; 2 = moderate; 3 = strong), ranging from 0 to 12. The score was evaluated by two independent pathologists, who determined that less than eight was considered to be low, and more than eight was considered to be high.

Cells were grown in a glass dish, fixed with 4% paraformaldehyde for 15 min, washed with PBS three times, blocked with blocking buffer (PBS with 5% BSA and 0.3% Triton X-100), and incubated overnight at 4°C with primary antibodies against BLM, γH2AX or p53 (1:100). The fluorescent secondary antibody (1:200) was incubated at 37 °C for 1 h. Cell nuclei were stained with DAPI (Sigma-Aldrich, Madrid, Spain).

### 4.6. Western Blotting (WB)

Cells were collected, lysed in RIPA buffer containing phenylmethanesulfonyl fluoride and phosStop, and centrifuged at 13,500× *g* at 4 °C for 30 min. After electrophoresing the proteins on 10% SDS-PAGE gels, the proteins were transferred onto polyvinylidene (PDVF) membranes (Millipore Corp., Birrica, MA, USA). Subsequently, the membranes were blocked, incubated with the primary antibody at 4 °C for 24 h, and incubated with the secondary antibody for 2 h. The membranes were then washed with TBST, and an ECL luminescent reagent (Beyotime, Shanghai, China) was added. GAPDH was used as an internal reference.

### 4.7. Molecular Docking Study

Docking studies were performed using Autodock 4.2 (http://autodock.scripps.edu/ downloads, accessed on 18 July 2020) and Autodock Vina (http://vina.scripps.edu/download.html, accessed on 25 July 2020) software. The crystal structure of BLM (code ID: 4CGZ) was used to construct the docking model. The adenosine diphosphate (ADP) in the crystal structure was removed. After removing duplicate molecules and filtering using Lipinski’s rule of five and Veber rules, all of the Specs database compounds were prepared using the program LigPrep module. Autodock Vina was used for the first round of docking. The top 1000 candidates were selected for further docking by the Autodock 4.2 software. The top 30 candidate compounds were purchased from a commercial supplier. The purity of these compounds was greater than 95%, as declared by the chemical supplier.

### 4.8. Expression and Purification of the BLM^642−1290^ Helicase

The recombinant E. coli pET-15b-BLM^642−1290^-BL21-CodonPlus was inoculated into LB media that contained 50 g/mL ampicillin and 30 g/mL chloramphenicol and cultured in a shaker incubator at 200 rpm at 37 °C until the optical density at 600 nm (OD600) reached 0.6. BLM^642−1290^ expression was then induced for 20 h (200 rpm, 18 °C) by 0.4 mM isopropyl-d-thiogalactoside (IPTG). The bacteria were then collected by centrifuging at 4000 rpm at 4 °C for 20 min. Next, the cells were lysed by a high-pressure cell crushing apparatus (Constant Systems Co., UK). The supernatant was collected by centrifugation at 13,000 rpm at 4 °C for 30 min (RC6-Puls, Thermo Co., Waltham, MA, USA). The BLM^642−1290^ was harvested after purification via nickel ion affinity chromatography and gel filtration chromatography (AKTA purifier 100, GE Healthcare Co., Marlborough, MA, USA). The purity of the BLM^642−1290^ product was assessed by bromophenol blue-stained 10% SDS-PAGE analysis and determined to be above 90%.

### 4.9. Fluorescence Polarization Assay

First, fluorescein-labeled dsDNA (2 nM) was added into a reaction buffer (20 mM Tris, 25 mM NaCl, 3 mM MgCl2, 0.1 mM DTT, pH 7.9) to detect the fluorescence anisotropy until it was stable. Then, 500 nM BLM^642−1290^ was added to equilibrate the DNA substrate, and the fluorescence anisotropy value was then detected. Finally, small molecules with a concentration of 10 μM were added, and the fluorescence anisotropy value was detected until it was stable. The total reaction volume was adjusted to 150 μL by adjusting with ddH_2_O volume. The ability of AO/854 to inhibit the ability of the BLM^642−1290^ to bind to dsDNA and ssDNA was also assessed using a fluorescence polarization assay. The method used was similar to that described above, except different concentrations of AO/854 were added to the reaction. By using the same protocol, we detected AO/854 affecting DNA unwinding of BLM^642−1290^. The 0–50 μM concentrations of AO/854 were added to the reaction. In the final step, 0.2 mM of ATP was added to the reaction. The fluorescence anisotropy value was detected until it was stable. The inhibiting ratio of BLM on DNA binding activity and DNA unwinding activity was determined [29].

### 4.10. ATPase Activity Analysis Assay

The malachite green-phosphate and ammonium molybdate colorimetry were used to detect the effect of AO/854 on the ATPase of BLM^642−1290^. AO/854, BLM^642−1290^ (125 nM), ssDNA (100 nM) and various AO/854 solutions (0–50 μM) were mixed into the reaction buffer respectively. The total reaction volume was adjusted to 75 μL by adjusting the ddH_2_O volume. The mixture was incubated at 25 °C for 10 min. ATP at a concentration of 2 mM was added and incubated for 20 min at 25 °C. The ATP hydrolysis was terminated by rapidly adding 50 μL of mixture to 850 μL of dye. One minute later, 100 μL of 34% citric acid solution was added to stop the color reaction. After that, a 100 μL mixture was added into one well of a 96-well. Three repeated wells were read at a length of 660 nm (Synergy H4). The enzyme amount was defined as an international unit. That is, a unit of enzyme is required to hydrolyze 1 μM of substrate per minute.

The computational formula for the enzymatic amount (units/mL) is as follows:

A: activity = 3B/10C

B: the standard curve’s calculated phosphate concentration (μM).

C: reaction time (minute).

The inhibiting ratio on ATPase activity of BLM was determined [29].

### 4.11. Electrophoretic Mobility Shift Assay (EMSA)

Fluorescence-labeled DNA (dsDNA, 100 nM) were added into the reaction buffer (20 mM Tris, 25 mM NaCl, 3 mM MgCl_2_, 0.1 mM DTT, pH 7.9). Next, different concentrations of AO/854 or ML216 (0-200 μM), BLM^642−1290^ (2.5 μM) and ATP (2 mM) were respectively added into the reaction buffer to start the DNA unwinding reaction. All reaction tubes were incubated for 2 h at 25°C. After 2 h, each tube received 100 μg/mL proteinase K to complete the reaction. The reaction complexes were electrophoresed in 0.5 × TBE on a 12% DNA retardation gel at 110V constant voltage for 2 h. The results were observed on the Bio-rad ChemiDoc Imaging System.

### 4.12. Unwinding Activity of Intracellular Assay

C2C12 mouse myoblast cells were seeded in special cell culture dishes and transfected for 24 h with GFP-RPA plasmids (2000 ng/μL) using Lipofectamine 3000 transfection reagent (Invitrogen, Termo Fisher), following the manufacturer’s instructions. After adding aphidicolin (50 μg/mL) and AO/854 or ML216 with different concentrations (0–15 μM), cells were imaged using a laser scanning confocal microscope every 5 min for 30 min. To measure and analyze the RPA accumulation, the GFP intensity inside each nucleus was measured for each time point, normalized to the GFP channel values at time point 0 (pretreatment), and the coefficient of variation of the GFP-channel intensities was plotted for each time point [30].

### 4.13. Circular Dichroism (CD) Assay

CD absorption spectra were recorded on a CD chromatograph at 25 °C (MOS-500, Bio-Logic, Seyssinet-Pariset, France). The CD curves were recorded as a blank for the correction of the PBS spectra. Next, a 10 μM concentration of BLM^642−1290^ was added into a series of 10 mL colorimetric tubes, and then different volumes of the AO/854 solution were added. At 25 °C, the CD absorption spectra in the wavelength range of 190–260 nm were detected. Each spectrum represented the average of three scans obtained by collecting data at a scan speed of 100 nm/minutes. The BLM^642−1290^ secondary structure was calculated using the DICRO 2000 software.

### 4.14. Cell Counting Kit-8 (CCK8) Drug Sensitivity Assessment and Cell Colony Formation Assay

The influence of AO/854 on the viability of PC3 cells was evaluated by the CCK8 (Saint-Bio, Shanghai, China) method. PC3 cells were plated at a density of 1 × 10^4^ in each well of a 96-well plate. After incubating for 24 h, the cells were treated with different concentrations of AO/854 or CDDP. The cells were then cultured for 24, 48, and 72 h, and then incubated with 10 μL of CCK8 at 37°C for 2 h. The automatic microplate reader (Synergy H4) was used to detect the OD450 value of each well. The inhibition ratio and IC_50_ (50% inhibiting concentration) of the drug on cell expansion were calculated using the OD values.

PC3 cells were seeded into 6-well plates at a density of 300/well. Different concentrations (0.5, 1, and 2 μM) of AO/854 were added into the wells. DMSO was used as a negative control. After culturing for 7 days, the cells were washed with PBS, fixed with methanol for 30 min, and stained with Giemsa for 30 min. The colony numbers were then counted.

### 4.15. Cell Cycle and Apoptosis Analysis

PC3 cells were seeded at a density of 1 × 10^5^ cells/well and allowed to grow for 24 h. Next, DMSO or AO/854 was added for an additional 48 h. Cells were then harvested and apoptosis was measured using an Annexin V/FITC Apoptosis Detection Kit (KeyGen Biotech, Jiangsu, China). The collected cells were mixed with 5 μL of FITC-Annexin V and incubated for 15 min at 25 °C in the dark. Next, 5 μL of propidium iodide (PI; BD Biosciences, San Jose, Cal., USA) was added and incubated for 5 min in the dark. Three independent replicates were analyzed by flow cytometer (Canto II Plus, BD Biosciences). For cell-cycle analysis, cells were digested with trypsin, collected, and fixed by adding 70% ethanol at 4 °C for 24 h. The cells were stained with PI for 15 min in the dark at 25 °C. The cell cycle was detected by a flow cytometer. All of the results were analyzed by FlowJoV10.

### 4.16. Transwell Assay and Scratch Wound-Healing Assay

For transwell assay, 100 μL of diluted atrigel (BD Biosciences) was added to the bottom center of the transwell chamber (Corning Inc., Corning, NY, USA) and incubated for 4 h at 37 °C. The cells were harvested, washed, and resuspended at a cell density of 1 × 10^6^ cells/mL. The cell suspension (200 μL) was added to the upper chamber, and complete medium containing 15% fetal calf serum was added to the lower chamber. After 10 h, the 23 atrigel and the cells in the upper chamber were removed, fixed with methyl alcohol for 20 min, and stained with Giemsa for 40 min. Five fields were randomly selected under the microscope to take pictures and count the number of cells invading the bottom of the chamber.

For scratch wound-healing assay, PC3 cells were plated into 6-well plates with complete medium and incubated overnight until they reached confluence. Wounds were created by using a 200 μL pipette tip. The plates were then washed with PBS to remove the scraped cells. Cells were treated with different concentrations of AO/854. Migration distance across the same scraped area was captured at 0 h (w1) and at 24 h or 48 h (w2). The percentage of wound closure was calculated as (w1 − w2)/w1 × 100%.

### 4.17. Alkaline Comet Assay

After drug treatment for 48h, cells were trypsinized, embedded in 1% low-melting agarose, and lysed in lysis buffer (10 mM Tris-HCl, 2.5 M NaCl, 100 mM EDTA, 1% Triton X-100 and 10% DMSO, pH 10.0) for 1 h. Slides were washed and incubated in cold electrophoresis buffer (300 mM NaOH, 1 mM EDTA) for 30 min. At the end, cells were stained with PI dye for 15 min. The free TriTek Comet Score software (TriTek Corp., Sumerduck, VA, USA) was used to quantify the images. At least 50 cells per sample were analyzed, and tail moment was determined as the percentage of DNA in the tail.

### 4.18. Nude Mouse Tumor Xenograft Model

Four-week-old male BALB/C nude mice were maintained under specific pathogen-free conditions in the animal facility for one week. Suspensions of PC3 cells (1 × 10^6^ viable cells/mouse) were subcutaneously injected into the right axillary of mice. Once the tumors reached approximately 100 mm^3^ in volume, six mice per group were randomized and treated with AO/854 (2 mg/kg body weight) or 0.9% saline by intraperitoneal injection every two days for 27 days. To evaluate the antitumor effect of the AO/854, tumors were measured with digital calipers every 3 days and volumes were estimated using the formula (length × width^2^)/2. Finally, the mice were euthanized, and the tumors were peeled off and weighed. The tumors were removed, fixed in paraffin, and sectioned for IHC and HE staining. IHC staining was used to examine protein expression, whereas HE staining was used to analyze structural alterations.

### 4.19. Quantitative Proteomics and Parallel Reaction Monitoring (PRM)

For quantitative proteomics, we extracted the total protein of PC3 cells, tested the quality of the protein, labeled peptides using TMT, separated fractions using a C18 column, obtained shotgun proteomics data using LC-MS/MS, and analyzed all proteomics data.

For PRM, we extracted the total protein of PC3 cells, tested the quality of the protein, digested it with trypsin, and obtained PRM data using LC-MS/MS [50,51].

### 4.20. Statistics and Analysis

For statistical analysis, SPSS software was used. The data were presented as the mean ± SD from three independent experiments. A Student’s *t*-test was used to assess differences between two groups. A one-way ANOVA was utilized when comparing multiple groups. Statistical significance was defined as a *p* value of less than 0.05. GraphPad Prism was used to analyze clinical data for survival and pertinent associations.

## 5. Conclusions

In summary, we identified a novel BLM inhibitor, named AO/854, via molecular docking virtual screening combined with an in vitro fluorescence polarization assay, a CD assay, and an EMSA study. The more intense cellular effects of AO/854 as compared ML216 suggested that the disrupting ability of compounds on BLM/DNA interaction might be more important in impacting BLM functions in cells. For the mechanism of action, AO/854 elicited DNA damage and triggered the DDR and downstream p53-dependent apoptosis in PC3 cells. On the basis of this, the identification of AO/854 provides a new perspective on the development of BLM in anti-prostate cancer treatment.

## Figures and Tables

**Table 1:** Relationships of BLM and clinicopathological characteristics in 60 patients with PCa.

Feature	N (60)	Immunohistochemical Score of BLM		χ^2^	*p* Value
		<8 (%)	≥8 (%)		
Age(years)	60	17	43	0.219	0.640
≤70	24	6 (25.0)	18 (75.0)		
>70	36	11 (32.4)	25 (67.6)		
Clinical stage	60	17	43	**10.506**	0.001 *
I–II	20	11 (55.0)	9 (45.0)		
III–IV	40	6 (15.0)	34 (85.0)		
Gleason score	60	17	43	2.694	0.101
<7	5	3 (60.0)	2 (40.0)		
≥7	55	14 (25.5)	41 (74.5)		
Gleason grade	60	17	43	**9.877**	0.002 *
1–3	15	9 (60.0)	6 (40.0)		
4–5	45	8 (17.8)	37 (82.2)		
N-regional lymph nodes	60	17	43	1.927	0.165
N0	43	10 (23.3)	33 (76.7)		
N1	17	7 (41.2)	10 (58.8)		
M-distant metastasis	60	17	43	0.410	0.522
M0	50	15 (30.0)	35 (70.0)		
M1	10	2 (20.0)	8 (80.0)		

* *p* < 0.05.

## Data Availability

All data generated or analyzed during this study are included either in this article or in the supplementary information files.

## Supplementary Materials

The supporting information can be downloaded at: https://www.mdpi.com/article/10.3390/ijms232314798/s1.
