# Supplementary material for: Discovery of a Novel Bloom’s Syndrome Protein (BLM) Inhibitor Suppressing Growth and Metastasis of Prostate Cancer
Source: Int J Mol Sci. 2022 Nov 26;23(23):14798. doi: 10.3390/ijms232314798 (PMC9736344; doi:10.3390/ijms232314798)
Supplement: Supplementary file 1 [file ijms-23-14798-s001.zip › Supplementary Materials/Table S1.docx]

Table S1.The Results of Molecular Docking

| **NO.** | **Ligand** | **Dock score using Auto**  **Dock (kcal/mol)** | **Dock score using Auto**  **Dock vina (kcal/mol)** | **Ligand interactions with 4CGZ** |
| --- | --- | --- | --- | --- |
| 1 | 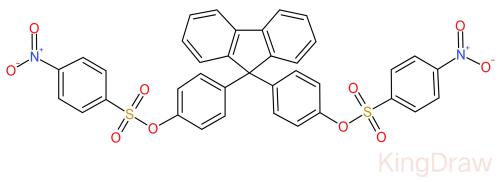 | -17.43 | -15.6 | 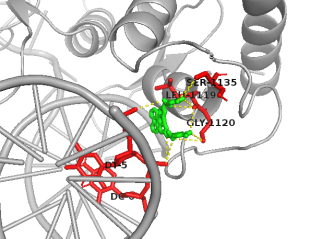 |
| 2 | 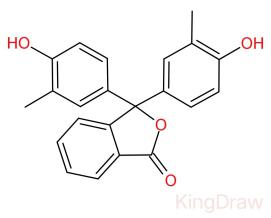 | -17.32 | -12.2 | 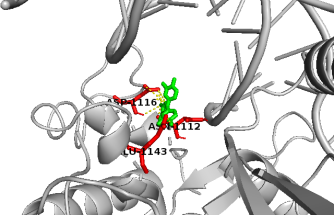 |
| 3 | 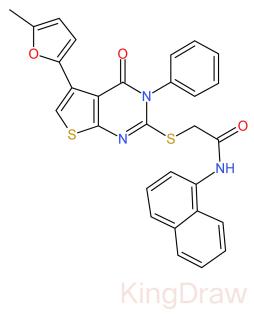 | -16.99 | -13.8 | 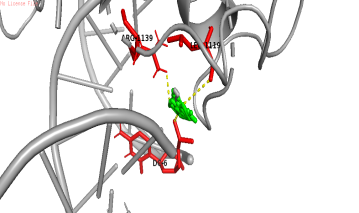 |
| 4 | 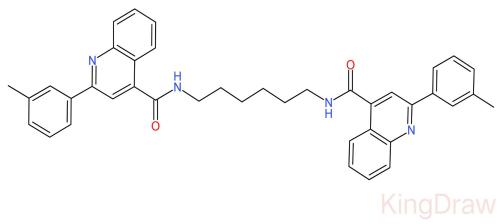 | -16.67 | -12.7 | 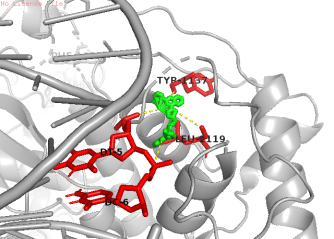 |
| 5 | 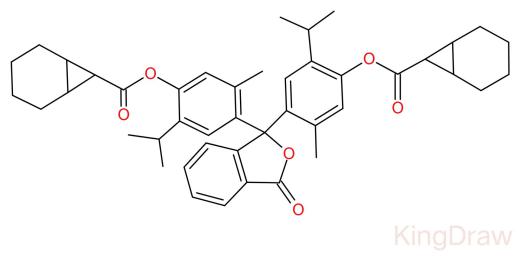 | -16.52 | -13 | 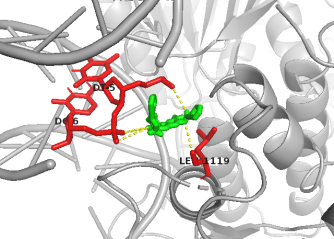 |
| 6 | 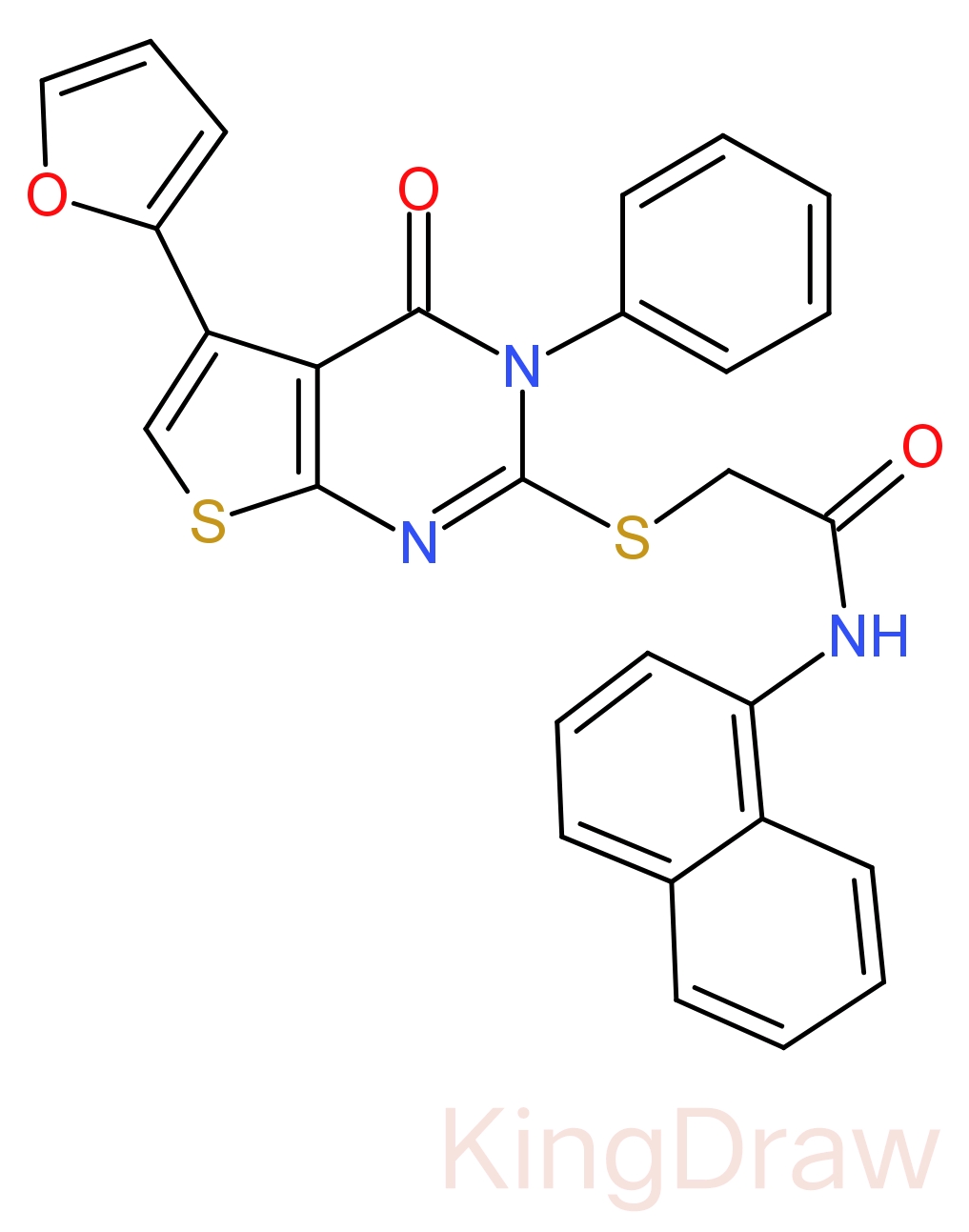 | -16.41 | -13.3 | 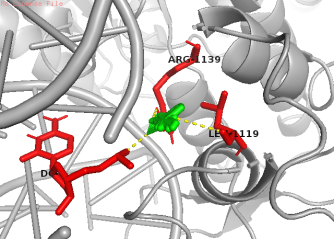 |
| 7 | 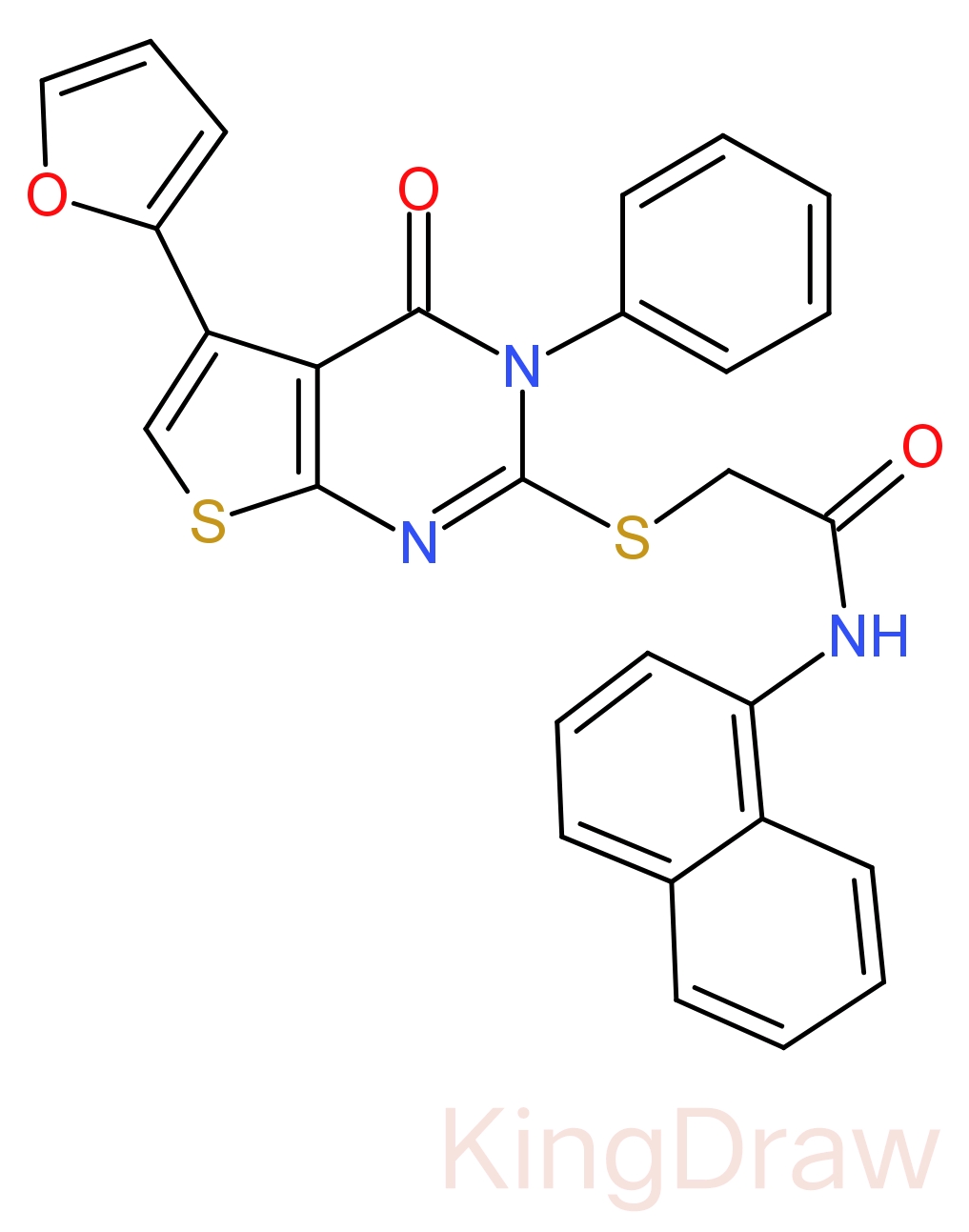 | -16.35 | -13.2 | 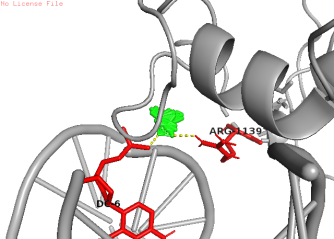 |
| 8 | 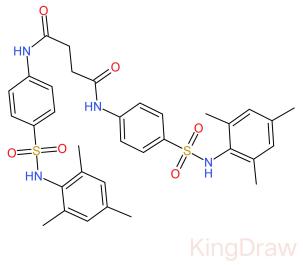 | -16.06 | -12.1 | 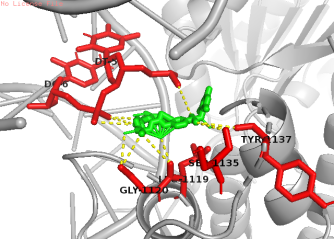 |
| 9 | 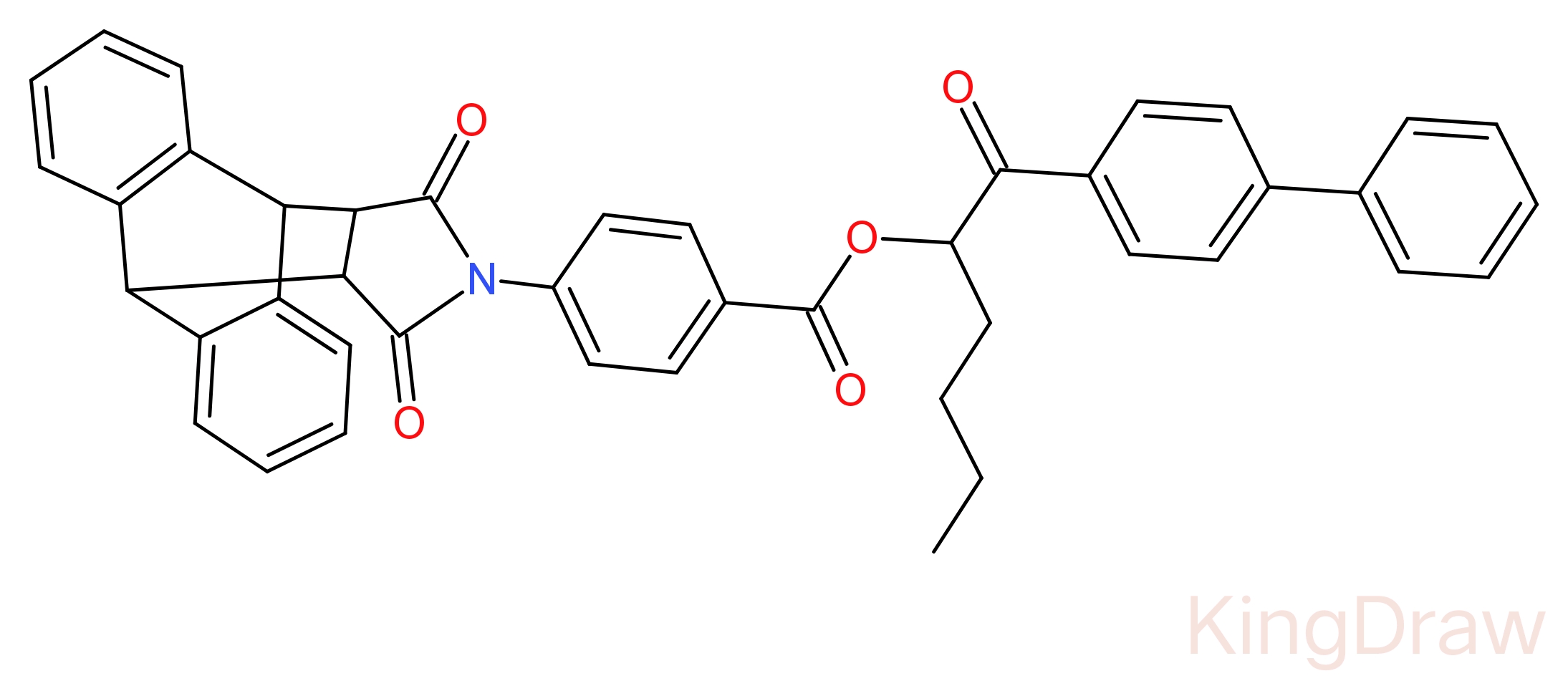 | -15.85 | -14.3 | 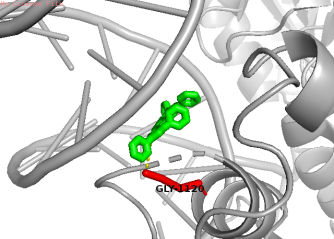 |
| 10 | 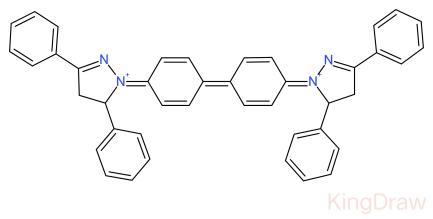 | -15.72 | -13.7 | 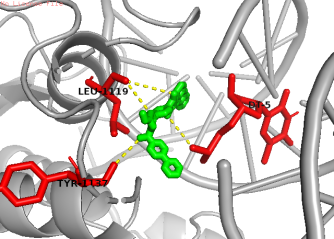 |
| 11 | 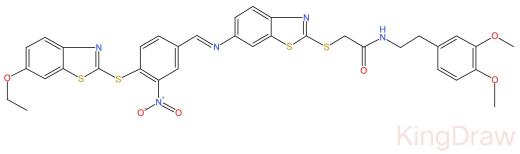 | -15.57 | -12 | 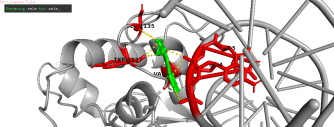 |
| 12 | 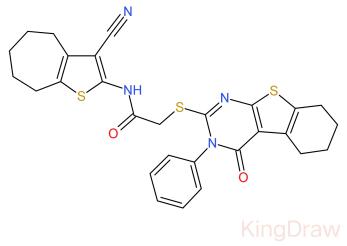 | -15.46 | -13.6 | 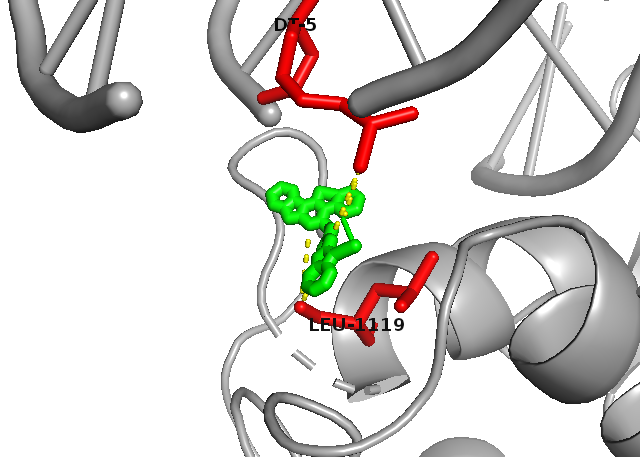 |
| 13 | 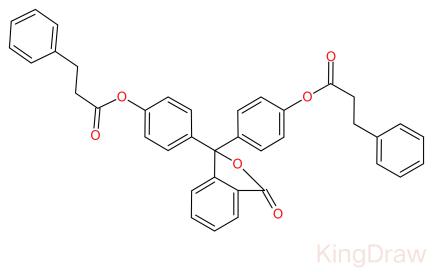 | -15.43 | -13.6 | 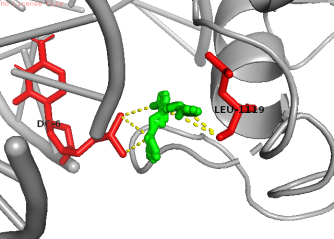 |
| 14 | 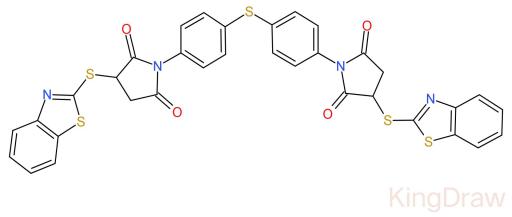 | -15.33 | -13.8 | 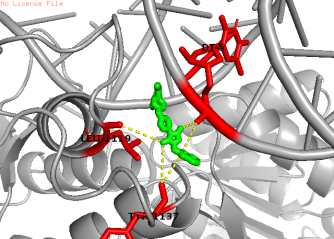 |
| 15 | 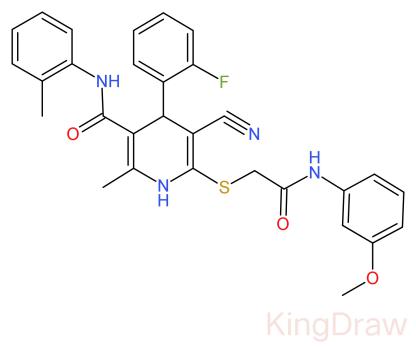 | -15.29 | -12.7 | 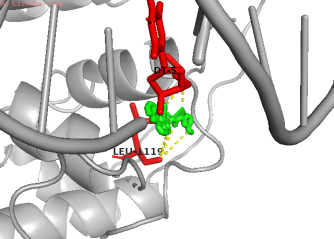 |
| 16 | 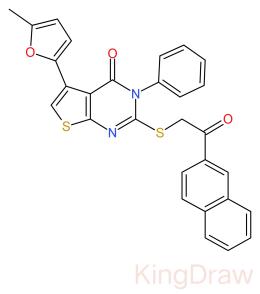 | -15.26 | -13.4 | 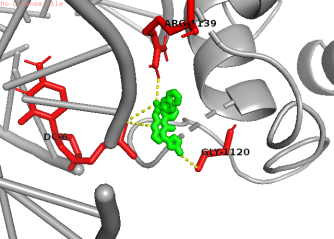 |
| 17 | 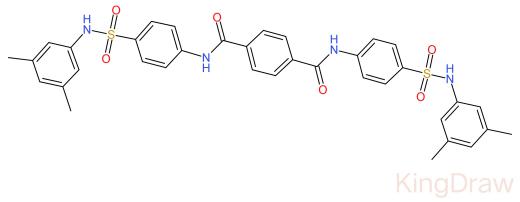 | -15.04 | -12.2 | 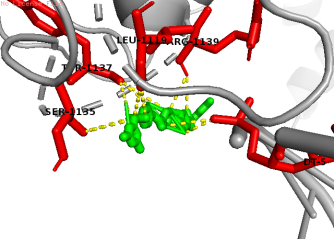 |
| 18 | 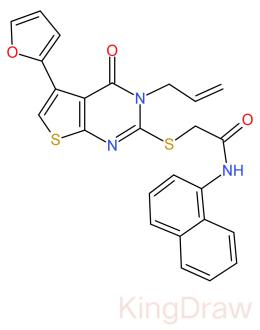 | -14.79 | -12 | 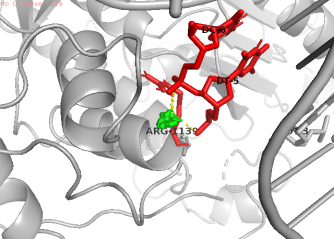 |
| 19 | 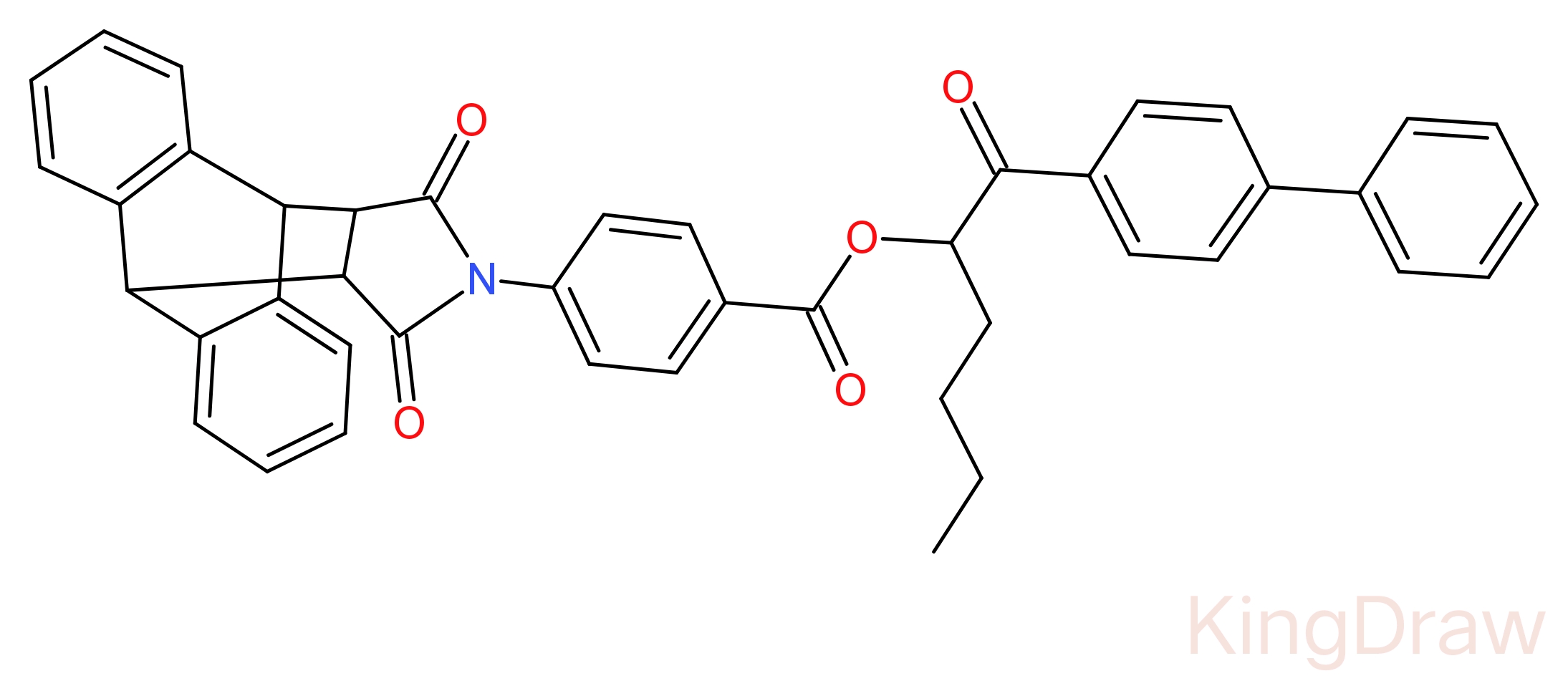 | -14.55 | -12 | 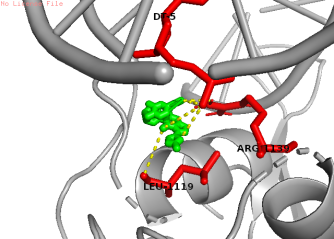 |
| 20 | 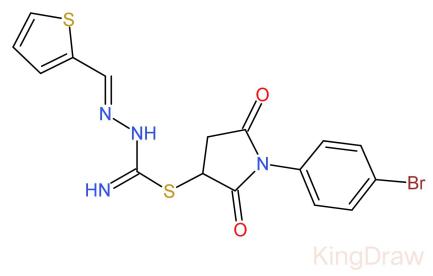 | -14.48 | -12.1 | 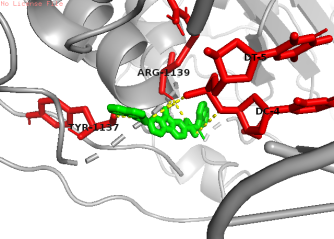 |
| 21 | 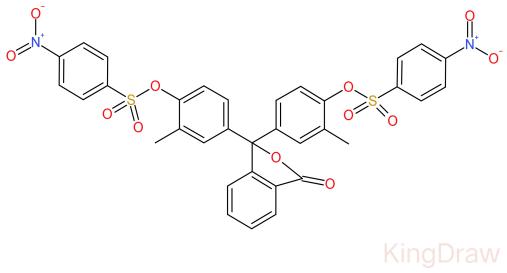 | -14.47 | -14.1 | 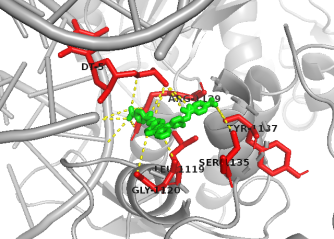 |
| 22 | 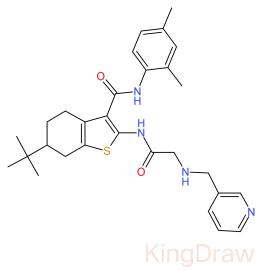 | -14.44 | -12.4 | 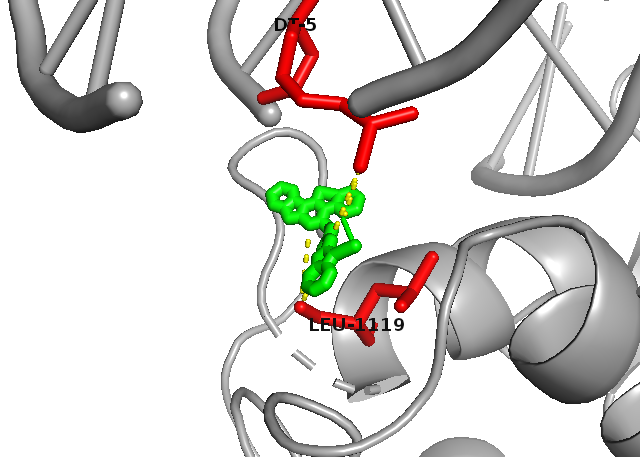 |
| 23 | 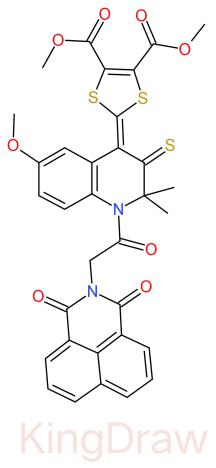 | -14.43 | -13.5 | 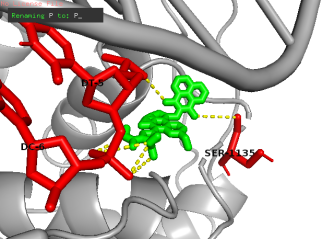 |
| 24 | 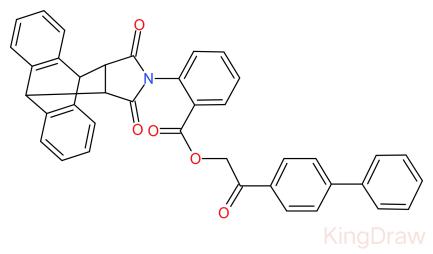 | -14.37 | -13.5 | 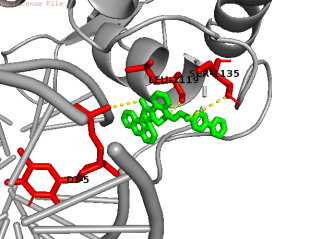 |
| 25 | 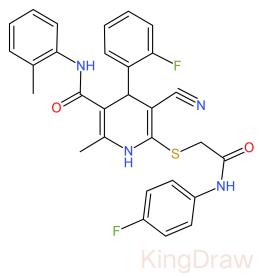 | -14.23 | -12.5 | 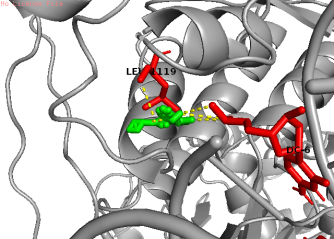 |
| 26 | 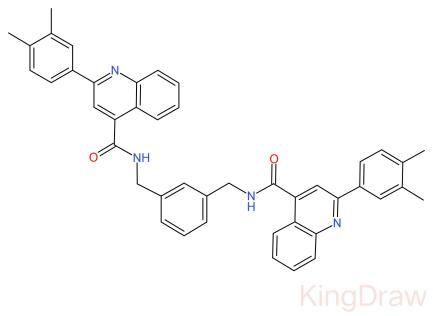 | -14.08 | -13.5 | 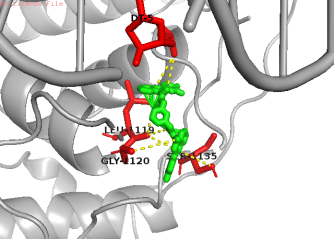 |
| 27 | 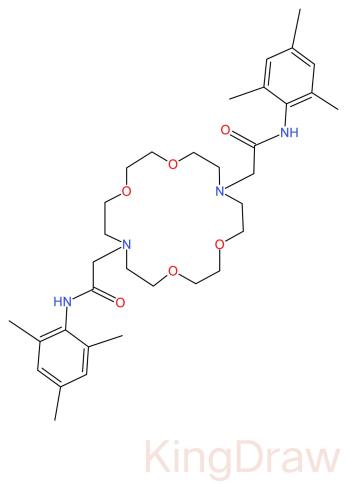 | -14.03 | -15.2 | 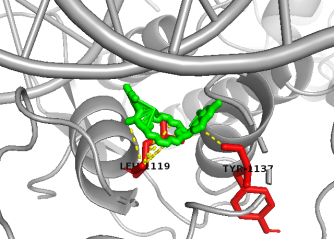 |
| 28 | 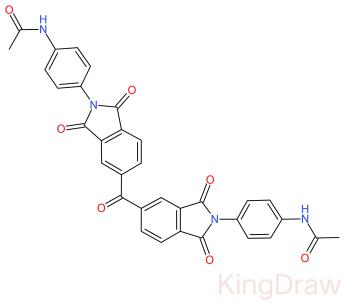 | -14.03 | -12.8 | 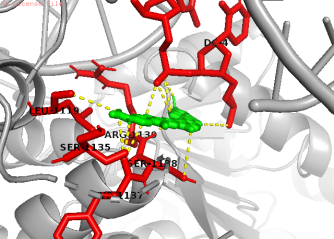 |
| 29 | 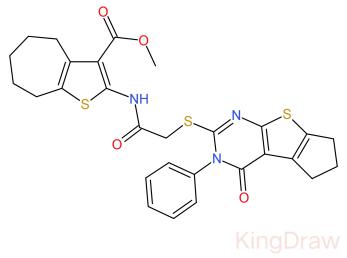 | -13.94 | -12.5 | 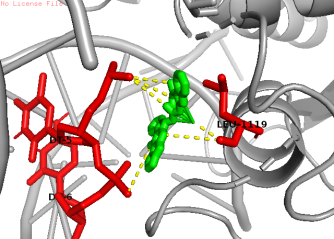 |
| 30 | 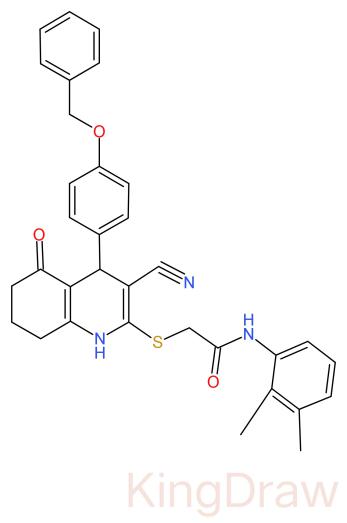 | -13.91 | -12.5 | 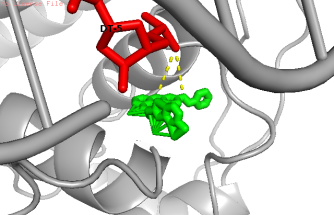 |
