# Supplementary material for: Discovery of a Novel Bloom’s Syndrome Protein (BLM) Inhibitor Suppressing Growth and Metastasis of Prostate Cancer
Source: Int J Mol Sci. 2022 Nov 26;23(23):14798. doi: 10.3390/ijms232314798 (PMC9736344; doi:10.3390/ijms232314798)
Supplement: Supplementary file 1 [file ijms-23-14798-s001.zip › Supplementary Materials/Table S2.docx]

Table S2 Effects of 30 compounds on DNA binding activity of BLM helicase

| **NO.** | **ID NO.** | **(A_20_-A_0_)±SD** | **Relative DNA binding activity/%** |
| --- | --- | --- | --- |
| 1 | AG-205/33006041 | -4.3±0.2 | 98.9 |
| **2** | **AO854/43447428** | **-268.1±2.8** | **31.5** |
| 3 | AF-399/40756801 | -19.9±0.8 | 94.9 |
| 4 | AK-968/40351464 | -7.7±0.2 | 98.0 |
| 5 | AN-329/40099095 | 4.2±0.1 | 101.1 |
| 6 | AF-399/40850930 | -67.3±1.8 | 82.6 |
| 7 | AF-399/40850931 | -46.2±1.1 | 88.2 |
| 8 | AN-329/40869136 | -5.4±0.1 | 98.6 |
| 9 | AE-848/30862027 | -1.8±0.05 | 99.5 |
| 10 | AK-918/12440383 | 10.3±0.2 | 102.7 |
| 11 | AG-205/11552188 | 2.1±0.05 | 100.5 |
| 12 | AF-399/40684381 | 8.3±0.2 | 102.2 |
| 13 | AO-856/40889317 | -14.4±1.2 | 96.3 |
| 14 | AK-968/40350116 | -7.7±0.4 | 98.0 |
| 15 | AE-848/41827296 | 1.2±0.01 | 100.3 |
| 16 | AF-399/40756933 | -4.7±0.2 | 98.8 |
| 17 | AN-329/40922909 | -13.5±0.8 | 96.6 |
| 18 | 18.AF-399/40850929 | -87±1.8 | 77.5 |
| 19 | AF-399/40756787 | -23.6±1.2 | 93.9 |
| 20 | AG-690/40104520 | -13.3±0.9 | 96.6 |
| 21 | AN-652/13689012 | -19.7±1.2 | 94.9 |
| 22 | AE-848/42024443 | 7±0.04 | 101.8 |
| 23 | AG-690/11837062 | -31.6±1.7 | 91.9 |
| 24 | AK-918/12272294 | -15.6±0.7 | 95.9 |
| 25 | AE-848/41827122 | 5.2±0.04 | 101.3 |
| 26 | AK-968/40351448 | -69.8±2.3 | 82.2 |
| 27 | AF-962/32160027 | 19.4±1.1 | 105.0 |
| 28 | AG-690/10800003 | -10.4±0.5 | 97.3 |
| 29 | AF-399/40851253 | -5.9±0.02 | 98.5 |
| 30 | AE-848/4093796 | -19.3±0.4 | 95.0 |

Note:A_0_ was the fluorescence anisotropy in the presence of the dsDNA and BLM helicase; A_20_ was the fluorescence anisotropy of the dsDNA, BLM helicase and AO/854 with the concentration of 20 μM.
