# Supplementary material for: Discovery of a Novel Bloom’s Syndrome Protein (BLM) Inhibitor Suppressing Growth and Metastasis of Prostate Cancer
Source: Int J Mol Sci. 2022 Nov 26;23(23):14798. doi: 10.3390/ijms232314798 (PMC9736344; doi:10.3390/ijms232314798)
Supplement: Supplementary file 1 [file ijms-23-14798-s001.zip › Supplementary Materials/Table S3.docx]

Table S3 The inhibition kinetics parameters of AO/854 on BLM^642-1290^ helicase

| Parameters | The concentration of AO/854 (µmol/L) | | |
| --- | --- | --- | --- |
|  | 0 | 10 | 20 |
| V_max_(µmol/L/min) | 2.7 | 2.6 | 2.7 |
| K_m_(mmol/L) | 0.24 | 1.8 | 4.3 |
| K_i_( µmol/L) | 1.37 | | |
| Type | competitive inhibition | | |
