# Supplementary material for: Discovery of a Novel Bloom’s Syndrome Protein (BLM) Inhibitor Suppressing Growth and Metastasis of Prostate Cancer
Source: Int J Mol Sci. 2022 Nov 26;23(23):14798. doi: 10.3390/ijms232314798 (PMC9736344; doi:10.3390/ijms232314798)
Supplement: Supplementary file 1 [file ijms-23-14798-s001.zip › Supplementary Materials/Table S4.docx]

Table S4 Differential proteins of PC3 cells after AO/854 treated

| **Protein_id** | **Description** | **Gene** | **B.vs.A FC** | **B.vs.A Pvalue** | **B.vs.A UP.DOWN** |
| --- | --- | --- | --- | --- | --- |
| P63261 | Actin, cytoplasmic 2 | ACTG1 | 0.6348 | 0.0128 | down |
| P26358 | DNA (cytosine-5)-methyltransferase 1 | DNMT1 | 0.7760 | 0.0385 | down |
| P52292 | Importin subunit alpha-1 | KPNA2 | 0.8251 | 0.0003 | down |
| T1R7A8 | MUC5AC (Fragment) | MUC5AC | 1.2048 | 0.0086 | up |
| P50995 | Annexin A11 | ANXA11 | 1.2267 | 0.0038 | up |
| A0A0C4DFL7 | Lanosterol 14-alpha demethylase | CYP51A1 | 1.2660 | 0.0012 | up |
| P09525 | Annexin A4 | ANXA4 | 1.2758 | 0.0068 | up |
| P56199 | Integrin alpha-1 | ITGA1 | 1.2158 | 0.0001 | up |
| Q96T88 | E3 ubiquitin-protein ligase UHRF1 | UHRF1 | 0.8206 | 0.0156 | down |
| P35908 | Keratin, type II cytoskeletal 2 epidermal | KRT2 | 1.6715 | 0.0039 | up |
| Q643R0 | HCTP4 | HCTP4 | 0.7863 | 0.0002 | down |
| Q4JM47 | AGR2 | AGR2 | 1.2090 | 0.0394 | up |
| A0A0A0MRM9 | Nucleolar and coiled-body phosphoprotein 1 (Fragment) | NOLC1 | 0.8317 | 0.0046 | down |
| Q14534 | Squalene monooxygenase | SQLE | 1.4513 | 0.0001 | up |
| Q96LJ7 | Dehydrogenase/reductase SDR family member 1 | DHRS1 | 1.2360 | 0.0008 | up |
| A0A6B7HGQ4 | MHC class I antigen | HLA-A | 1.2420 | 0.0040 | up |
| P07996 | Thrombospondin-1 | THBS1 | 1.2155 | 0.0044 | up |
| A0A0S2Z4Z6 | Serine/arginine repetitive matrix 1 isoform 2 (Fragment) | SRRM1 | 0.8212 | 0.0199 | down |
| Q99685 | Monoglyceride lipase | MGLL | 0.8006 | 0.0027 | down |
| Q9H8V3 | Protein ECT2 | ECT2 | 0.8016 | 0.0373 | down |
| B5MBX0 | Sororin | CDCA5 | 0.7939 | 0.0002 | down |
| B4DEE8 | 6-O-methylguanine-DNA methyltransferase | MGMT | 0.8142 | 0.0193 | down |
| Q5TZN3 | UBE2C protein | UBE2C | 0.8025 | 0.0105 | down |
| A8K3S3 | Kinesin-like protein |  | 0.8074 | 0.0042 | down |
| Q9BWH2 | FUN14 domain-containing protein 2 | FUNDC2 | 1.3301 | 0.0284 | up |
| Q3LIE7 | Delta(24)-sterol reductase | Nbla03646 | 1.4657 | 0.0005 | up |
| Q15004 | PCNA-associated factor | PCLAF | 0.7388 | 0.0160 | down |
| P02768 | Albumin | ALB | 1.2188 | 0.0496 | up |
| Q8WYB3 | Beta II spectrin-short isoform (Fragment) |  | 0.8130 | 0.0170 | down |
| Q99988 | Growth/differentiation factor 15 | GDF15 | 1.2819 | 0.0003 | up |
| Q14512 | Fibroblast growth factor-binding protein 1 | FGFBP1 | 1.2718 | 0.0006 | up |
| Q8IYA6 | Cytoskeleton-associated protein 2-like | CKAP2L | 0.8118 | 0.0412 | down |
| A0A286YFF7 | Palmitoyl-protein hydrolase 1 | PPT1 | 1.2085 | 0.0026 | up |
| Q9Y6A9 | Signal peptidase complex subunit 1 | SPCS1 | 1.2223 | 0.0247 | up |
| A0A024R2I7 | RING-type E3 ubiquitin transferase RAD18 | RAD18 | 0.8153 | 0.0204 | down |
| A0A024R832 | Anaphase-promoting complex subunit CDC26 | CDC26 | 1.3645 | 0.0018 | up |
| Q8NBM8 | Prenylcysteine oxidase-like | PCYOX1L | 1.2245 | 0.0067 | up |
| Q96GM5 | SWI/SNF-related matrix-associated actin-dependent regulator of chromatin subfamily D member 1 | SMARCD1 | 0.8260 | 0.0068 | down |
| A6NGJ0 | Dynein light chain Tctex-type 3 | DYNLT3 | 1.2165 | 0.0325 | up |
| B4DSL6 | cDNA FLJ57190, highly similar to Actin-binding protein anillin |  | 0.7992 | 0.0325 | down |
| A8K2A0 | cDNA FLJ76770 |  | 1.2299 | 0.0140 | up |
| A0A0A6YY92 | Adenylosuccinate lyase | ADSL | 1.2709 | 0.0022 | up |
| Q6P453 | Ubiquitin carboxyl-terminal hydrolase (Fragment) | USP11 | 1.4217 | 0.0489 | up |
| Q9Y548 | Protein YIPF1 | YIPF1 | 1.2523 | 0.0131 | up |
| Q9BX90 | Magphinin beta | TRO | 1.3135 | 0.0016 | up |
| A0A678ZXX5 | MHC class I antigen | HLA-C | 1.2032 | 0.0197 | up |
| B4DFJ7 | Tetraspanin 31, isoform CRA_a | TSPAN31 | 1.2105 | 0.0019 | up |
| Q6PK81 | Zinc finger protein 773 | ZNF773 | 1.2004 | 0.0122 | up |
| Q9BPW9 | Dehydrogenase/reductase SDR family member 9 | DHRS9 | 1.2431 | 0.0013 | up |
| C9J0W7 | Adiponectin receptor protein 1 (Fragment) | ADIPOR1 | 1.3389 | 0.0003 | up |
| O60635 | Tetraspanin-1 | TSPAN1 | 1.2143 | 0.0014 | up |
| Q14525 | Keratin, type I cuticular Ha3-II | KRT33B | 0.8125 | 0.0495 | down |
| K4DI92 | RWD domain containing 4A | RWDD4 | 1.2151 | 0.0321 | up |
| Q8NCF5 | NFATC2-interacting protein | NFATC2IP | 0.8298 | 0.0068 | down |
| P20248 | Cyclin-A2 | CCNA2 | 0.8333 | 0.0477 | down |
| Q6PIK7 | Angiogenic factor with G patch and FHA domains 1 | AGGF1 | 2.2335 | 0.0084 | up |
| P15514 | Amphiregulin | AREG | 1.3800 | 0.0121 | up |
| O15392 | Baculoviral IAP repeat-containing protein 5 | BIRC5 | 0.7924 | 0.0009 | down |
| A0A024QZN5 | ZW10 interactor, isoform CRA_b | ZWINT | 0.8105 | 0.0187 | down |
| Q9NYZ3 | G2 and S phase-expressed protein 1 | GTSE1 | 0.8006 | 0.0376 | down |
| P23511 | Nuclear transcription factor Y subunit alpha | NFYA | 0.8252 | 0.0017 | down |
| A0A2R8YCV2 | Uncharacterized protein |  | 1.3282 | 0.0016 | up |
| B7Z7S9 | cDNA FLJ61724, highly similar to Shugoshin-like 2 |  | 0.8230 | 0.0013 | down |
| A0A0A0MR04 | Signal peptidase complex catalytic subunit SEC11C | SEC11C | 1.3777 | 0.0029 | up |
| Q86V24 | Adiponectin receptor protein 2 | ADIPOR2 | 1.3152 | 0.0053 | up |
| A0A2R8Y7G1 | Aryl hydrocarbon receptor | AHR | 0.7628 | 0.0001 | down |
| U3KX66 | ATP synthase protein 8 | ATP8 | 1.2297 | 0.0289 | up |
| B4DWN4 | Nuclear envelope phosphatase-regulatory subunit 1 |  | 1.2440 | 0.0038 | up |
