# Supplementary material for: Discovery of a Novel Bloom’s Syndrome Protein (BLM) Inhibitor Suppressing Growth and Metastasis of Prostate Cancer
Source: Int J Mol Sci. 2022 Nov 26;23(23):14798. doi: 10.3390/ijms232314798 (PMC9736344; doi:10.3390/ijms232314798)
Supplement: Supplementary file 1 [file ijms-23-14798-s001.zip › Supplementary Materials/Table S5.docx]

Table S5 Relative expression of target proteins in PRM

| **NO.** | **Protein_id** | **A1** | **A2** | **A3** | **B1** | **B2** | **B3** |
| --- | --- | --- | --- | --- | --- | --- | --- |
| 1 | Q4JM47 | 0.04277 | 0.0723 | 0.0861 | 0.0871 | 0.0799 | 0.0902 |
| 2 | Q96LJ7 | 0.00271 | 0.0031 | 0.0022 | 0.0032 | 0.0032 | 0.0035 |
| 3 | P26358 | 0.00315 | 0.0041 | 0.0028 | 0.0021 | 0.0020 | 0.0025 |
| 4 | A8K3S3 | 0.00079 | 0.0014 | 0.0010 | 0.0006 | 0.0006 | 0.0006 |
| 5 | A0A0A0MRM9 | 0.00604 | 0.0070 | 0.0053 | 0.0042 | 0.0043 | 0.0050 |
| 6 | A0A0C4DFL7 | 0.00464 | 0.0058 | 0.0051 | 0.0069 | 0.0072 | 0.0087 |
| 7 | P63261 | 0.10524 | 0.1472 | 0.1525 | 0.1356 | 0.1038 | 0.1223 |
| 8 | B5MBX0 | 0.00123 | 0.0014 | 0.0012 | 0.0006 | 0.0005 | 0.0007 |
| 9 | A0A0S2Z4Z6 | 0.00073 | 0.0010 | 0.0010 | 0.0006 | 0.0006 | 0.0006 |
| 10 | P56199 | 0.00156 | 0.0023 | 0.0021 | 0.0025 | 0.0025 | 0.0027 |
| 11 | Q99988 | 0.00024 | 0.0003 | 0.0003 | 0.0007 | 0.0007 | 0.0009 |
| 12 | Q643R0 | 0.00446 | 0.0028 | 0.0019 | 0.0025 | 0.0015 | 0.0017 |
| 13 | Q3LIE7 | 0.00261 | 0.0029 | 0.0025 | 0.0048 | 0.0045 | 0.0068 |
| 14 | Q96GM5 | 0.00050 | 0.0007 | 0.0006 | 0.0004 | 0.0003 | 0.0005 |
| 15 | P09525 | 0.00247 | 0.0032 | 0.0021 | 0.0034 | 0.0032 | 0.0041 |
