# Supplementary figures and images for: Discovery of a Novel Bloom’s Syndrome Protein (BLM) Inhibitor Suppressing Growth and Metastasis of Prostate Cancer
Source: Int J Mol Sci. 2022 Nov 26;23(23):14798. doi: 10.3390/ijms232314798 (PMC9736344; doi:10.3390/ijms232314798)

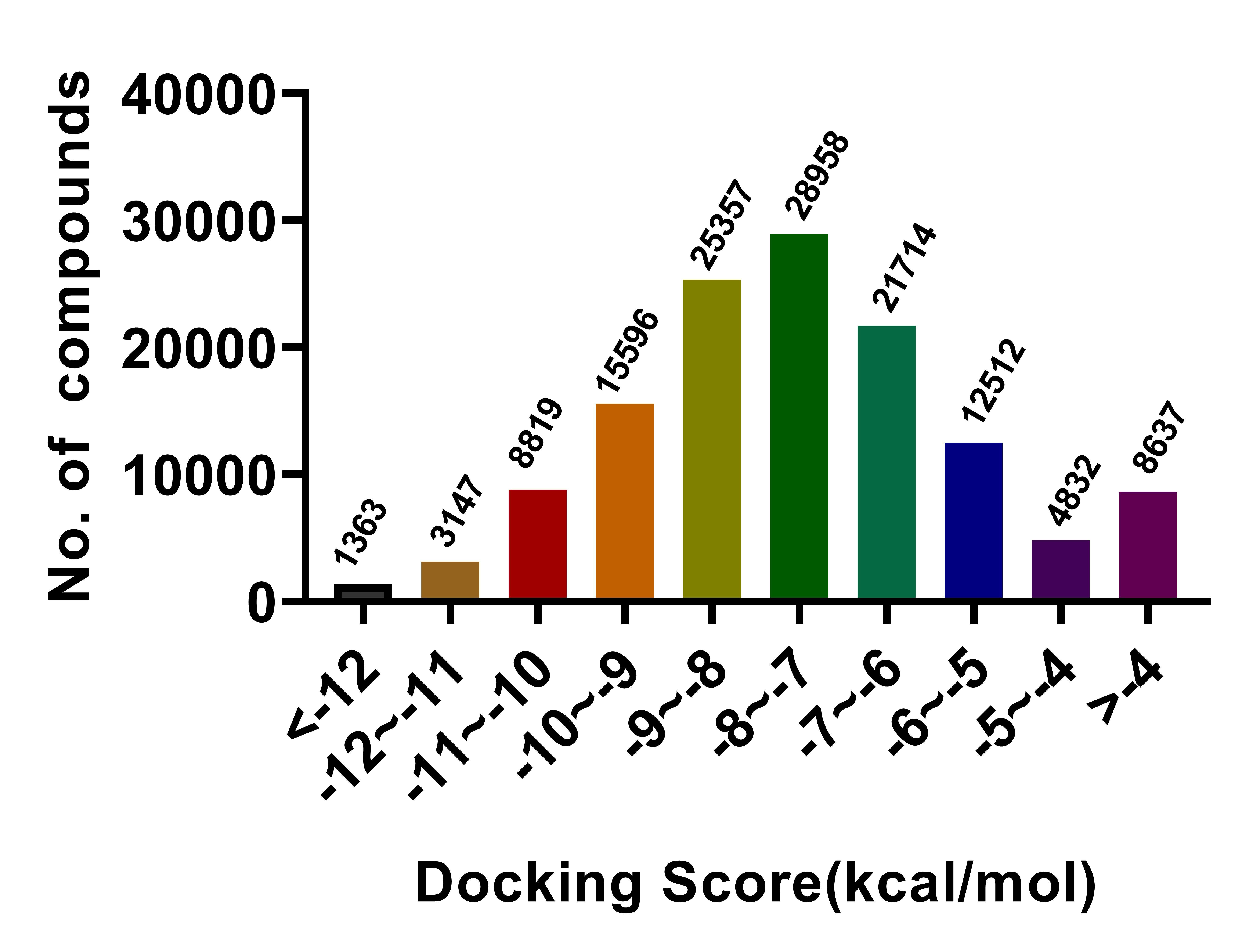

Supplement: Supplementary file 1 [file ijms-23-14798-s001.zip › Supplementary Materials/Figure S1.tif]

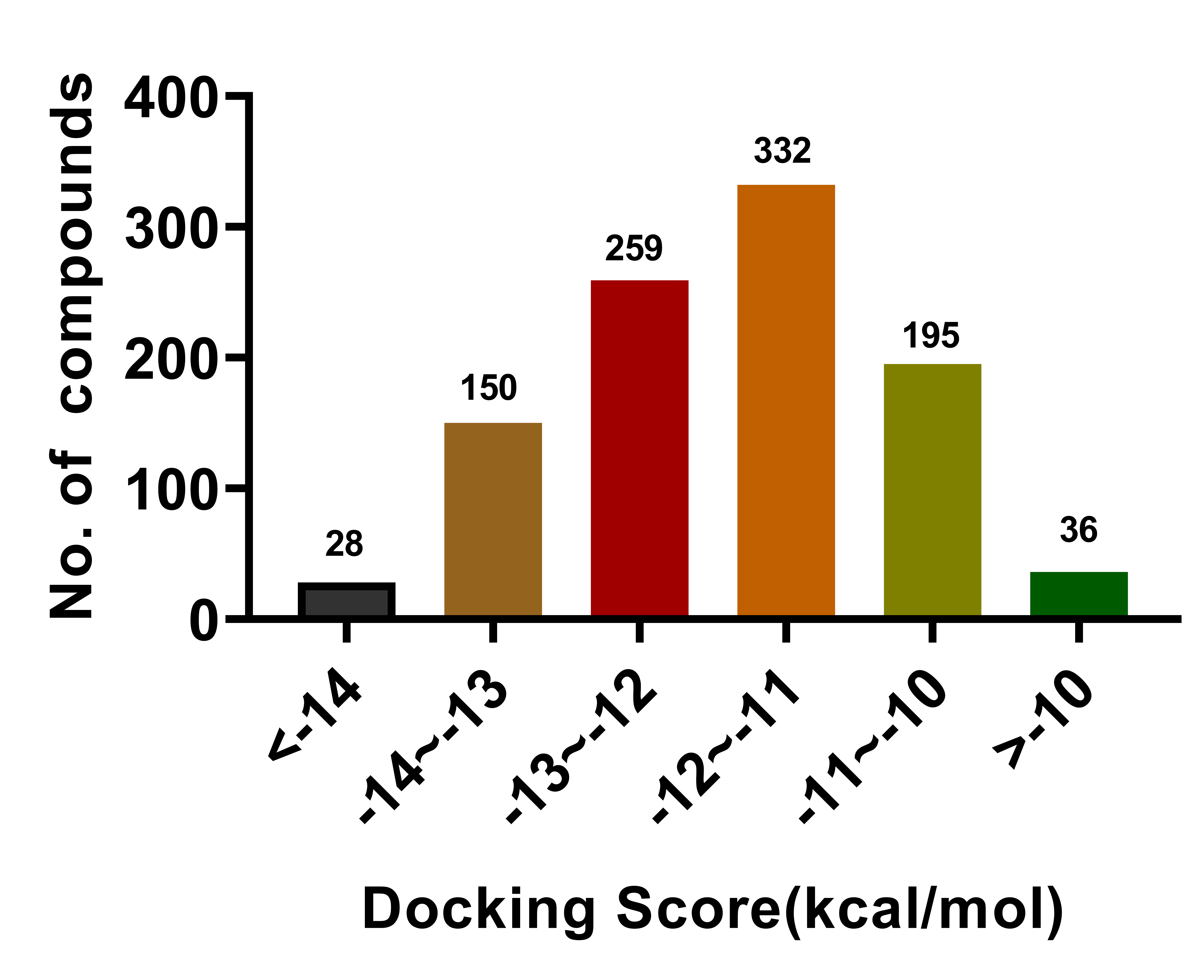

Supplement: Supplementary file 1 [file ijms-23-14798-s001.zip › Supplementary Materials/Figure S2.tif]

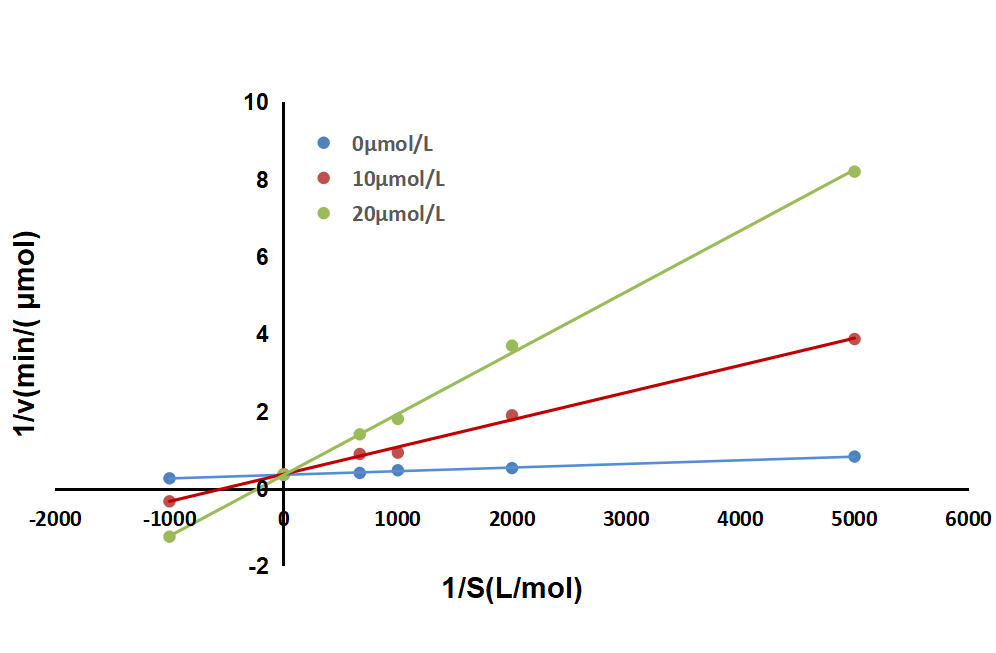

Supplement: Supplementary file 1 [file ijms-23-14798-s001.zip › Supplementary Materials/Figure S3.tif]

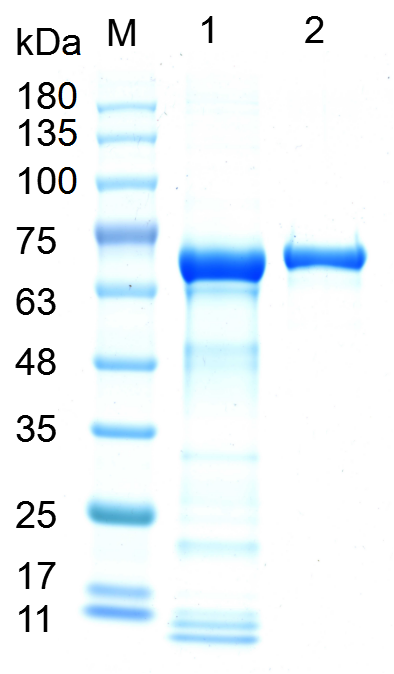

Supplement: Supplementary file 1 [file ijms-23-14798-s001.zip › Supplementary Materials/Figure S4.tif]

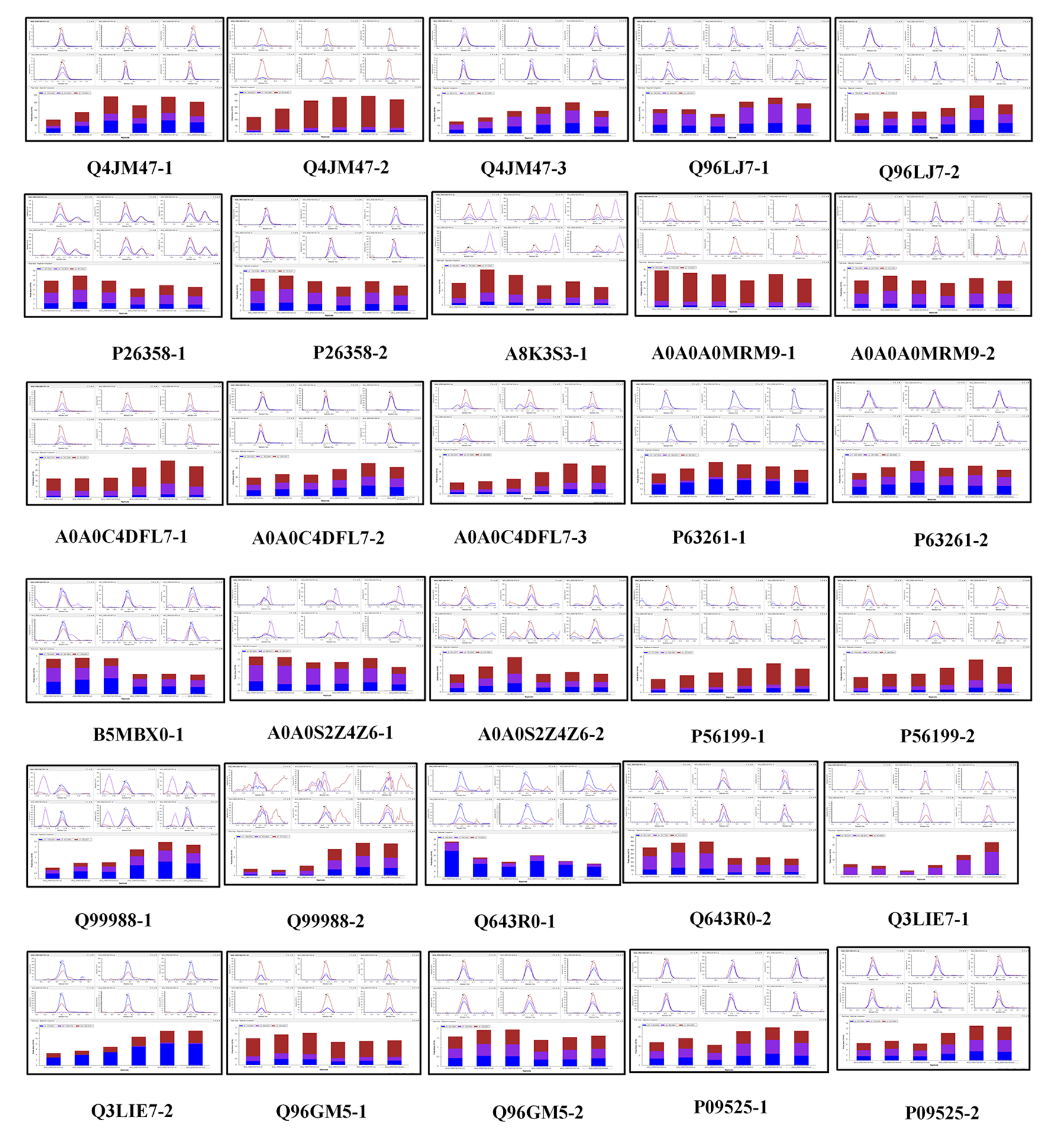

Supplement: Supplementary file 1 [file ijms-23-14798-s001.zip › Supplementary Materials/Figure S5.tif]
